# The Missing Link: Connecting Cultivation Conditions and Refolding Performance via Inclusion Body Biophysical Properties

**DOI:** 10.1002/bit.70033

**Published:** 2025-08-05

**Authors:** Matthias Rüdt, Aymerick Bussien, Joan Cortada‐Garcia, Holger Morschett, Hauke Holm, Chiara Mazzucchelli, Karlheinz Flicker

**Affiliations:** ^1^ Institute of Life Sciences, School of Engineering HES‐SO University of Applied Sciences and Arts Western Delémont Switzerland; ^2^ Lonza AG Visp Switzerland

**Keywords:** biophysical characterization, cultivation conditions, design of experiments, inclusion bodies, protein refolding, spectroscopy, statistical modeling

## Abstract

Inclusion bodies (IBs) frequently form during the expression of heterologous proteins in *Escherichia coli* and are therefore important in pharmaceutical production. While it is accepted that cultivation conditions affect cultivation performance, the link to refolding performance remains unexplored. This study proposes that this interplay relies, at least partially, on the inherited biophysical properties of the IBs. Using a design of experiments approach, this study systematically explored how cultivation conditions—postinduction temperature, pH, and feed rate—affect the production of IBs containing anti‐desipramine single‐chain variable fragment antibodies as a model therapeutic protein. Various biophysical properties of the IBs—including hydrophobicity, secondary structure, and particle size—were characterized, and their relationship to cultivation parameters and refolding performance was analyzed. Key findings revealed that higher feed rates and temperatures increased the product titer and IB size. Larger IBs facilitated refolding, while a higher content of amyloid structures, occurring locally without a strong link to the cultivation parameters, hampered protein solubilization and refolding efficiency. Higher protein content in the IBs adversely affected the refolding yield due to a hidden coupling between cultivation and refolding. This study establishes IB biophysical properties as critical factors for linking upstream and refolding process performance, offering actionable insights to enhance bioprocess robustness and efficiency.

## 1 Introduction

About one‐third of the market‐approved pharmaceutical products made in cell‐based systems are produced in microbial expression systems, particularly in *Escherichia coli* (*E. coli*) (Walsh and Walsh 2022). Despite potential performance advantages during cultivation (Demain and Vaishnav 2009), heterologous expression in microbial systems often leads to the formation of insoluble protein aggregates known as inclusion bodies (IBs) (Bhatwa et al. 2021). To convert IBs into pharmaceutically active ingredients, they must be solubilized and refolded into their native structure. Established IB solubilization and refolding processes such as dilution refolding typically require large refolding tanks and consume big amounts of chaotropic and redox agents as well as water (Freydell et al. 2007; Mannall et al. 2007). This leads to a high plant footprint and material consumption, ultimately translating into high costs and environmental burdens. Thus, improving IB processes could positively impact the economics and sustainability of biopharmaceutical production.

As IBs are protein aggregates, it is imperative to consider their biophysical properties, such as the particle size and structure: Larger IBs are generally desired as they have a lower surface‐to‐volume ratio, reducing surface‐bound impurities like membrane proteins, nucleic acids, and lipids. They are also considered less susceptible to degradation by host proteases (García‐Fruitós et al. 2012; Rinas et al. 2017; Valax and Georgiou 1993; Ventura and Villaverde 2006). Moreover, larger IBs facilitate easier recovery via centrifugation due to higher terminal settling velocities (Vallejo and Rinas 2004).

There is increasing evidence that IBs are not simply composed of randomly assembled, unstructured polypeptide chains (“boiled egg structure”), but instead contain two distinct higher‐order structural elements (Rinas et al. 2017): a porous backbone composed of an amyloid‐like structure and native proteins integrated into the porous backbone. The former consists of stacked β‐sheets which give IBs a dense, chemically inert structure and their characteristic resistance to chemical, enzymatic, and physical degradation (Elia et al. 2017; Singh et al. 2020; Wang et al. 2008). The latter can retain a fraction of their activity (Rinas et al. 2017; Singh et al. 2015; Singh and Panda 2005; Ventura and Villaverde 2006).

Cultivation parameters, including pH, temperature, medium, carbon availability, and induction parameters, have been shown to significantly influence the ratio of soluble to insoluble protein production and IB properties such as size, secondary structure, impurity profile, and potential refolding performance (Hernandez 2013; Castellanos‐Mendoza et al. 2014; Fahnert et al. 2004; Gutiérrez‐González et al. 2019; Peternel et al. 2008; Slouka et al. 2019; Valax and Georgiou 1993). However, altering cultivation conditions can negatively impact overall bioprocess performance. Therefore, to maximize the recovery of active protein from IBs, it is essential to understand how cultivation conditions affect both the product titer and the biophysical properties, and how these different structural properties affect IB refolding performance. Other studies have followed a systematic Design of Experiments (DoE) approach to understand the relationship between refolding performance and certain process parameters such as chromatography parameters (Kateja et al. 2017), or DTT concentration and solubilization time (Ebner et al. 2021). However, the relationship between multivariate cultivation conditions and process performance, especially refolding yield linked to the biophysical IB attributes, has not been studied systematically.

This study employs DoE to investigate the effects of three key cultivation parameters—postinduction temperature, pH, and feed rate—on the production of an anti‐desipramine antibody single‐chain fragment variable (scFv) in the form of IBs in *E. coli*. The study further examines the multivariate effect of these cultivation conditions on IB biophysical properties. Finally, the link between these IB properties and refolding yields is explored.

## 2 Materials and Methods

### 2.1 Bacterial Strain and Media

All cultivations were carried out using a proprietary industrial *E. coli* Lonza XS strain (Lonza AG), derived from BL21 genetic background. It carries a plasmid for the expression of an antibody scFv, predominantly in the form of IBs. Expression was controlled by the rhamnose promoter (*P*_*rhaB*_). The cryopreservation medium consisted of peptone yeast extract (PYE) containing 10 g kg^‐1^ glucose and 50 µg mL^‐1^ kanamycin. Proprietary industrial cultivation semi‐defined medium (Lonza AG) was used for all pre‐cultures and main cultivations.

### 2.2 Buffers

Buffers as presented in Table 1 are prepared from deionized H_2_O. Where needed, pH was adjusted using 1 M NaOH or 1 M HCl. Buffers were stored at room temperature.

**Table 1:** Composition of buffers utilized throughout the study.

Buffer	Components	pH
Resuspension buffer	25 mM Tris‐HCl, 10 mM EDTA	8.0
IB wash buffer	25 mM Tris‐HCl, 10 mM EDTA, 2 M urea	8.0
IB solubilization buffer	8 M guanidine‐HCl, 20 mM Tris (TRIZMA base), 5 mM EDTA	8.4
IB refolding buffer	4 M urea, 50 mM Tris, 2 mM l‐cystine, 5 mM l‐cysteine	7.0
Dialysis buffer	20 mM Tris, 150 mM NaCl	7.0
SDS buffer	100 mM Tris‐HCl, 1% SDS	9.0
SEC buffer	150 mM NaCl and 100 mM NaH_2_PO_4_	6.8
SEC storage buffer	150 mM NaCl, 100 mM NaH_2_PO_4_, 0.05% (w v^‐1^) NaN_3_	6.8

### 2.3 Experimental Design of the Cultivation Conditions

The experimental design was developed in the JMP Statistical Discovery software (version 17.0.0, SAS Institute, Cary, North Carolina, USA) and included a total of 24 cultivations distributed in two blocks of 12 parallel bioreactor runs. Three factors ‐ pH, temperature, and feed rate ‐ were varied. The factor ranges were fixed based on historical process knowledge. The pH was adjusted in the range of 6.2–7.5; temperature varied between 18°C and 35°C. The feed rate range is presented in standardized, pseudonymized form to ensure consistency while safeguarding sensitive information. All remaining process parameters were kept at standard operating conditions. The center point was inserted as a unicate and cultivation conditions of relevance for process development were added as triplicate. The remaining 20 experiments were distributed by maximizing the determinant of the information matrix $X^{\prime }X$ resulting in a constrained D‐optimal design (de Aguiar et al. 1995; Eriksson et al. 2008). The experimental design matrix is given in Supporting Information S1: Table S3.

### 2.4 Cultivation

While cultivation methods are detailed in the following sections, a schematic summary is shown in Supporting Information S1: Figure S1. Strain maintenance is described in Supplementary section 1.1.

#### 2.4.1 Precultures

Pre‐cultures were prepared by inoculating 100 mL of semi‐defined medium (see Section 2.1) in threefold baffled 500 mL shake flasks to an initial OD_600_ of 0.01 with cryoculture (see Supplementary section 1.1) and incubated at 35°C, 150 rpm orbital shaking and 25 mm shaking diameter for 13 h. The cultures were harvested during the exponential phase (approx. OD_600_ 3) to inoculate the main cultures.

#### 2.4.2 Main Cultivation Process

All main cultivations were carried out in an Ambr 250 High Throughput (Sartorius) automated cultivation platform, which allowed up to 12 pre‐sterilized single‐use stirred tank reactors (STRs) to be run in parallel. These STRs feature an agitator shaft with two Rushton impellers, an electrochemical probe for inline pH measurement, and an optical sensor sport for inline dissolved oxygen (DO) measurement. Aeration was supplied using thermal mass flow controllers and off‐gas online analyzed for O_2_ and CO_2_ content per STR. The STRs were filled with 110 mL of semi‐defined medium (see Section 2.1) and inoculated to an initial OD_600_ of 0.10 from the same preculture (see Section 2.4.1). All STRs were run at 35°C and 1 v v^−1^ min^−1^ submersed aeration with compressed air. Based on probe readout, pH was titrated to 7.0 using 7.5% (w w^−1^) H_2_SO_4_ and 12.5% (w w^−1^) NH_4_OH. DO was maintained at ≥ 30% using a cascaded control loop (1000–4500 rpm stirring speed, 0–150 mL min^−1^ additional submersed aeration with oxygen). After an initial batch phase (typically 10.5–11.0 h), an exponential feed with a set growth rate of 0.15 h^−1^ was applied for another 12 h for further biomass amplification. For the subsequent production phase, feed rate, pH, and temperature were reactor‐specifically adjusted as per the experimental design (see Section 2.3). Cultures were induced by aseptic rhamnose addition to 1 g L^−1^ final concentration 2 h into the production phase. The production phase lasted 46 h after induction, at which point the cells were harvested by 45 min centrifugation at $4.2\cdot 10^{3}g$ and 4°C. The supernatants were discarded, and the harvested cell pellets stored at −80°C.

### 2.5 Cell Lysis and IB Recovery

Harvested cells at the end of the fermentation process were lysed and IBs were recovered from the insoluble fraction as detailed below. A schematic illustration of the workflow of IB recovery, protein solubilization, and refolding can be found in Supporting Information S1: Figure S2.

#### 2.5.1 Cell Lysis and Dry Cell Weight Determination

The frozen pellets (see Section 2.4‐2) were thawed and resuspended to a concentration of 200 g L^−1^ biomass wet weight in resuspension buffer by 1 h constant magnetic stirring with a 4 cm stir bar at 600 rpm. Before cell lysis, the cell dry weight (CDW) was determined by drying a 5 mL aliquot of the resuspended wet biomass in a halogen scale (HR83 Halogen, Mettler Toledo, Switzerland) at 105°C. Resuspended cells were lysed in a French press high‐pressure homogenizer (Model SPCH‐10, Stansted, United Kingdom) by processing 45 mL of cell suspension for three cycles with pressure maintained within 950 to 1050 bar. Homogenized material was collected in a Falcon tube placed in an ice water bath to prevent sample overheating.

#### 2.5.2 IB Washing

Crude lysates were centrifuged at $1.5\cdot 10^{4}g$ and 4°C for 20 min. The resulting supernatant (soluble fraction) was collected in 1 mL aliquots and frozen at −80°C until further analysis. The pellet (insoluble fraction) was resuspended in 40 mL IB wash buffer and incubated for 1 h at RT. Following the incubation period, the material was centrifuged at $1.5\cdot 10^{4}g$ and 4°C for 15 min; this process was repeated for three cycles to remove impurities. The pellet was resuspended in 40 mL IB wash buffer and centrifuged. The supernatant was discarded, and the pellets (washed IBs) were weighed for mass balance. Six aliquots of 20 mg washed IBs per experimental condition were prepared and frozen at −80°C in Eppendorf tubes. The remaining washed IB pellets were frozen at −80°C, freeze‐dried, and later used for the biophysical characterization (see Section 2.8).

### 2.6 IB Solubilization and Refolding

In Supporting Information S1: Figure S2, the workflow of IB recovery, protein solubilization, and refolding are visualized in a flow chart. The experiments were conducted in four blocks of six samples each.

#### 2.6.1 Protein Solubilization

For each of the 24 experimental conditions, two 20 mg aliquots of washed IBs were thawed and resuspended in 82.6 µL IB solubilization buffer. The Eppendorf tubes were placed in a tube rotator (Stuart, Cole‐Parmer) and mixed at 60 rpm for 15 min. Following the initial incubation, 2.1 μL 1 M DTT solution was added resulting in a final concentration of 20 mM DTT to reduce disulfide bonds. The solution was incubated in the tube rotator for an additional 45 min. The two tubes were pooled and mixed by pipetting. Half of the sample was analyzed by size exclusion chromatography (SEC, see Section 2.7.2) and the other half was subjected to protein refolding (see Section 2.6.2).

#### 2.6.2 Protein Refolding

The solubilized protein was transferred into 15 mL tubes. Over 120 min, 9.9 mL IB refolding buffer was added to each tube in eight separate additions to simulate a dilution‐refolding protocol with continuous buffer feeding, reaching a final concentration of 0.20 g L^−1^ based on the solubilized IB mass. The tubes were sparged with nitrogen for 15 min, sealed air‐tight and incubated at 4°C without agitation for 21 h. Afterwards, the tubes were sparged with air for 15 min and incubated without caps (open tubes) for 16 h at 4°C to allow for the oxidation of disulfide bridges. The tubes were centrifuged at $1.5\cdot 10^{4}g$ and 4°C for 20 min to remove precipitate. The clarified soluble protein was further processed by dialysis in a 4°C cold room to minimize protein degradation. For the dialysis for each refolding experiment, a 500 mL beaker was filled with 400 mL of cold dialysis buffer. A dialysis cassette (Slide‐A‐Lyzer Dialysis Cassettes 3.5 K MWCO, Thermo Scientific) was hydrated for 5 min in cold dialysis buffer. Then, 3 mL of the protein solution was injected into the dialysis cassette. The protein solution was dialyzed for 2 h and mixed at 100 rpm by magnetic stirring with a 4 cm stir bar. After 2 h, the 400 mL of dialysis buffer was replaced with 400 mL of fresh cold dialysis buffer and dialysis was continued overnight for 21 h total duration. The protein solutions were removed from the cassettes using a syringe, and filtered to remove precipitate with 0.45 µm cellulose acetate (CA) filters.

### 2.7 Analytics

#### 2.7.1 Capillary Gel Electrophoresis (CGE)‐SDS

Analyses were conducted on a PA800 plus (ScieX, Canada) equipped with a bare fused silica capillary (50 µm internal diameter, 30 cm length, 20 cm capillary length to detector, ScieX) and a photodiode array detector acquiring at 280 nm.

The internal standard was prepared by dissolving 25 mg of chicken egg lysozyme (AppliChem, product ID: A4972) in 5 mL of desalted water. The effective concentration was determined with a cuvette absorbance measurement (Ultrospec 2100 Pro, Biochrom Ltd, UK), at 280 nm assuming an absorption coefficient of 2.65 mL mg^‐1^ cm^‐1^ as obtained from the Expasy ProtParam tool (Gasteiger et al. 2005) for the non‐reduced form of Lysozyme from *Gallus gallus* (UniProt ID P00698). A 20 mg IB aliquot was thawed and solubilized in 1 mL 10% (w v^−1^) SDS solution for 1 h at 37°C in a ThermoMixer C incubator (Eppendorf) set to 500 rpm. A size standard was prepared by mixing 90 µL SDS buffer with 5 µL MW size standard (SDSMW analysis kit, ScieX), 5 µL 2‐mercaptoethanol (BME), and 2 µL internal standard. Blanks were prepared by mixing 95 µL SDS buffer with 5 µL BME and 2 µL internal standard. The samples were prepared by mixing 20 µL solubilized IBs with 75 µL SDS buffer, 5 µL BME, and 2 µL internal standard. All preparations were incubated for 10 min at 70°C.

After pre‐conditioning of the capillary, the samples were injected with 5 kV for 20 s. Separation was performed with 15 kV for 30 min. Absorbance traces were collected at 280 nm and evaluated as detailed in Supplementary section 1.2 providing the scFv mass fraction per washed wet IB pellet and the scFv mass fraction per CDW.

#### 2.7.2 SEC HPLC

Analyses were conducted on a 1260 Infinity II HPLC device (Agilent) equipped with a TSKgel UP‐SW2000 15 cm column (TOSOH Bioscience) and a DAD acquiring from 220 to 400 nm. Before use, the column was equilibrated for 1 h with SEC buffer at 0.35 mL min^‐1^ flow rate at room temperature. For analysis, a 10 µL sample (0.45 µm CA‐filtered) was injected and run for 8 min at 0.35 mL min^‐1^ with SEC buffer. To be consistent with the CE‐SDS method, concentrations were calculated based on the theoretical molar extinction coefficient of the scFv (${ɛ}_{P}\;=\;51.59{\;\text{kL mol}}^{-1}{\text{cm}}^{-1}$) obtained from the Expasy ProtParam tool (Gasteiger et al. 2005) for the non‐reduced scFv.

### 2.8 Biophysical Characterization

All samples for biophysical characterization were withdrawn from the washed IB pellet (see Section 2.5‐2) and, except for the laser diffraction (LD) measurements, freeze‐dried at −80°C and 25 Pa for 24 h (Cryodos, Telstar, Spain) and comminuted (if necessary) with a mortar and pestle before further treatment.

#### 2.8.1 Raman Spectroscopy

Raman measurements were performed in triplicate using an inVia InSpect confocal Raman microscope (Renishaw, UK). Measurements were carried out with a 785 nm NIR laser, with a 100x short distance objective (high NA: 0.85) with 10 s of exposure time at 100% laser power. Spectra from 100 to 3200 cm^−1^ were recorded.

#### 2.8.2 FT‐IR Spectroscopy

The FT‐IR measurements were performed on a Nicolet iS50 FTIR Spectrometer (Thermo Fisher Scientific, USA) equipped with a germanium attenuated total reflectance (ATR) unit. For each spectrum, 16 scans with 4 cm^−1^ resolution were averaged using the automatic atmospheric suppression function of the instrument. For a measurement, freeze‐dried IB powder was dispensed onto the ATR crystal, compressed with the ATR unit lever onto the crystal and the measurement was initiated.

#### 2.8.3 Fluorescence Spectroscopy

The scoop of a micro spatula (a few mg) was filled with freeze‐dried washed IB powder (see Section 2.8) and transferred to a 1.5 mL Eppendorf tube. The dry powder was resuspended in 1 mL of desalted water. 200 µL of the suspension was dispensed into a 96‐well UV transparent flat‐bottom microtiter plate (Corning, USA). The emission spectrum from 300 to 370 nm was acquired with a SpectraMax iD3 reader (Molecular Devices, USA) for three excitation wavelengths (260, 270, and 280 nm) via bottom reading with “low” gain settings. The plate was automatically agitated orbitally for 5 s at “medium” shaking intensity between each excitation wavelength run.

#### 2.8.4 Laser Diffraction

100 mg of washed wet IBs were defrosted and resuspended in 2 mL ultrapure water by pipetting. This suspension was then 5 µm PES filtered (Macherey‐Nagel, Germany) and directly dispensed in the measurement well of a FLOWSYNC laser diffractor (Microtrac MRB). The following parameters were set before starting the analysis: 20 s set zero‐time, 60 s run time, 3 runs, transparent particles, 1.5 refractive index of particles, irregular shape, water as fluid, 1.333 refractive index of fluid with sonication for particle dispersion. The median diameter was recorded for further analysis.

### 2.9 Data Processing and Analysis

Data analysis and processing was performed in Python 3.9.18 (Python Software Foundation, USA) using Jupyter Notebooks with Jupyter Lab 4.0.8 (Kluyver et al. 2016). Matrix and data frame handling was performed with NumPy 1.26.0 (Harris et al. 2020) and pandas 2.1.3 (McKinney 2010). Visualizations were generated with matplotlib 3.8.3 (Hunter 2007) and seaborn 0.13.2 (Waskom 2021). The environment definition, the code, and the data are published as open research data (Zenodo: https://doi.org/10.5281/ZENODO.16315733).

#### 2.9.1 Statistics

For elucidating the relations between factors and responses, ordinary least squares (OLS) regression and generalized linear models (GLM) were used as implemented in the statistics library statsmodels 0.14.0 (Seabold and Perktold 2010). Factors were normalized before regression and a naïve, global model was used as a starting point. The model was optimized by minimizing the corrected Akaike Information Criterion (AICc) through the elimination of irrelevant factors (Burnham and Anderson 2002). Whenever the process parameters served as model factors, a linear‐quadratic model with interaction terms was used as a global model. The higher‐order model was selected since a process optimum close to the center of the experimental design was expected. The response was modeled as directly linear with OLS regression.

For the correlation of the biophysical attributes with the mass fraction of product in the IB and the refolding yield, a GLM model with normal error and a logarithmic linker function was used. All biophysical characteristics were considered as linear factors only. The performance of the best GLM model was evaluated during 10‐fold cross‐validation with 100 repeats as implemented in scikit‐learn 1.3.2. The relative root mean squared error of cross validation (RMSECV) was normalized by the maximum of each response. The pseudo *R* ^2^ was calculated as described by Cox and Snell (1989), which compares the likelihood of a constant model against the likelihood of the used model. The prominence of the AICc indicates how much better the best model is compared to the following.

#### 2.9.2 Analysis of Spectroscopic Data

Raman and FT‐IR spectra were pre‐processed through a first normalization with scikit‐learn 1.3.2 (Pedregosa et al. 2011), asymmetric Whittaker baseline subtraction with chemometrics 0.4.0 (Rüdt 2022) and a second normalization. The first normalization allowed to improve the consistency of the subsequent baseline subtraction.

To reduce the dimensionality of the spectroscopic data, the spectra were factorized by principal component analysis (PCA). The number of principal components (PCs) was selected with a scree plot and the observed spectral noise in the loadings.

## 3 Results and Discussion

**Figure 1:**
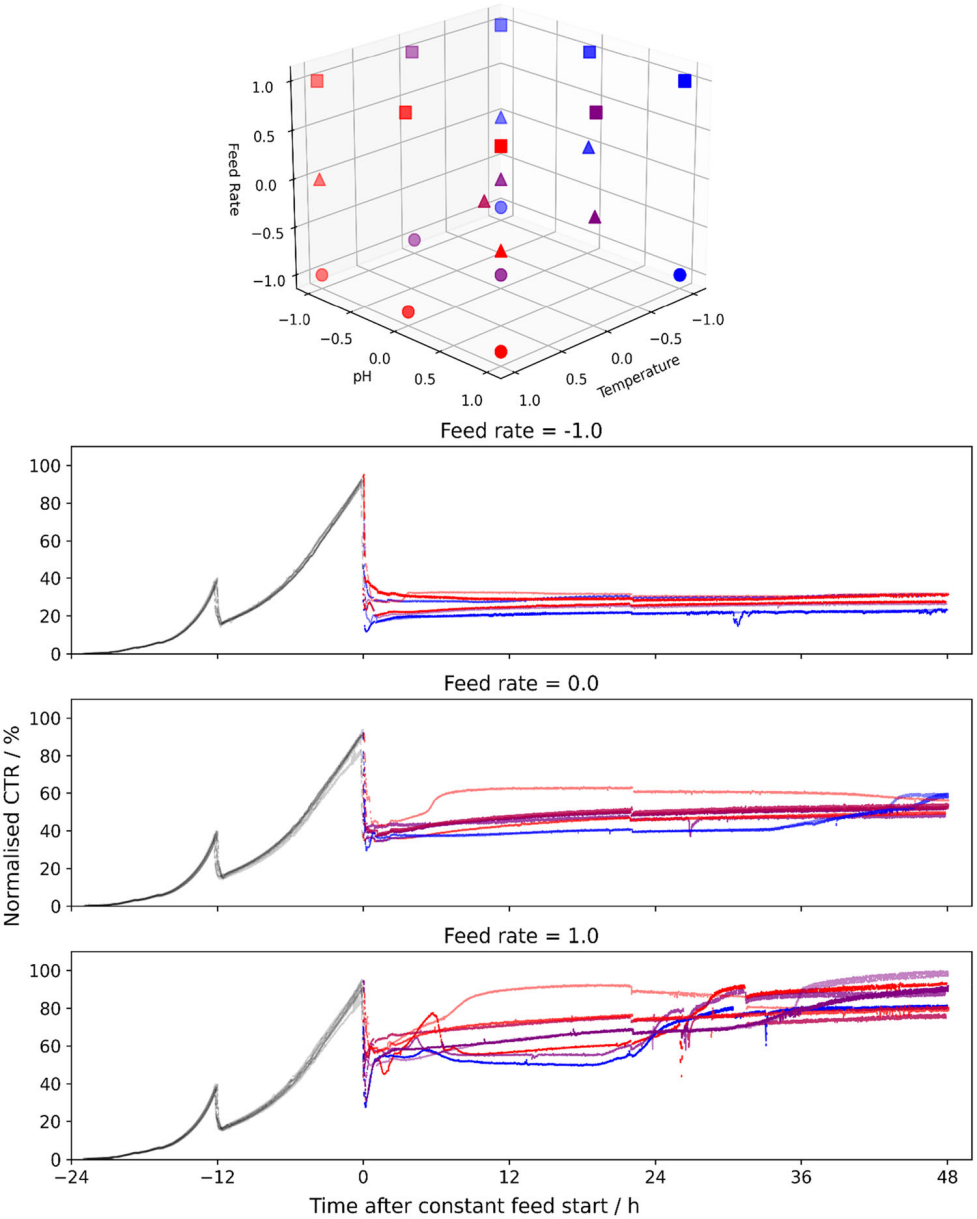
Top: D‐Optimal design of the cultivation parameters investigated for their impact on IB formation. Temperature is colored from blue (low) to red (high); pH is marked with high and low opacity; feed rate is marked with distinct markers. Bottom: CTR trajectories for all cultivations, colored in gray until the start of production phase (time 0 h). From this point, the temperature and pH are indicated by color and opacity as mentioned above. The different feed rates are shown in different subplots. To simplify visualization, the cultivation at −0.03 normalized feed rate is not shown.

Variations of an industrially‐relevant model process were performed to explore the impact of upstream process conditions on IB formation, IB biophysical properties, and corresponding refolding performance. Cultivation parameters were selected and varied based on their expected impact on IB quality and refolding yield: Employing a semi‐quantitative rating scheme, parameters were chosen based on literature reports and existing process expertise and three key process parameters—pH, temperature, and feed rate during induction—were selected for further investigation (Supporting Information S1: Table S2). Subsequently, these parameters formed the factors of a d‐optimal experimental design (Figure 1 top), guiding the variation of process conditions and enabling the evaluation of their effects on upstream process performance (see Section 3.1), IB biophysical properties (see Section 3.2) and refolding performance (see Section 3.4).

### 3.1 Effect of Process Parameters on Cultivation Key Performance Indicators

A total of 24 cultivations were performed in two blocks of 12 parallel cultivations. During the biomass propagation phase, all cultivations maintained identical process conditions. The carbon transfer rate (CTR) (Figure 1 bottom), an indicator of metabolic activity, confirms highly comparable trajectories across all cultivations. On the other hand, diverging metabolic responses are reflected in the CTR patterns of the production phase where variance was introduced as per the d‐optimal‐design. Already at this point, such a response gives strong evidence that the chosen parameters are influential and changes in cultivation key performance indicators (KPIs) are to be expected.

From a process development point of view, the most interesting conditions are the ones where high cell densities alongside high product mass fractions and purities are achieved, ultimately leading to the highest final product mass. Therefore, the four KPIs selected to characterize the upstream performance were: (1) the total CDW to evaluate how much biomass can be obtained until harvest, (2) the mass fraction of product per CDW as an indicator of cellular productivity, (3) the purity of the IBs to assess the product content of the IBs, and (4) the total product mass generated.

**Figure 2:**
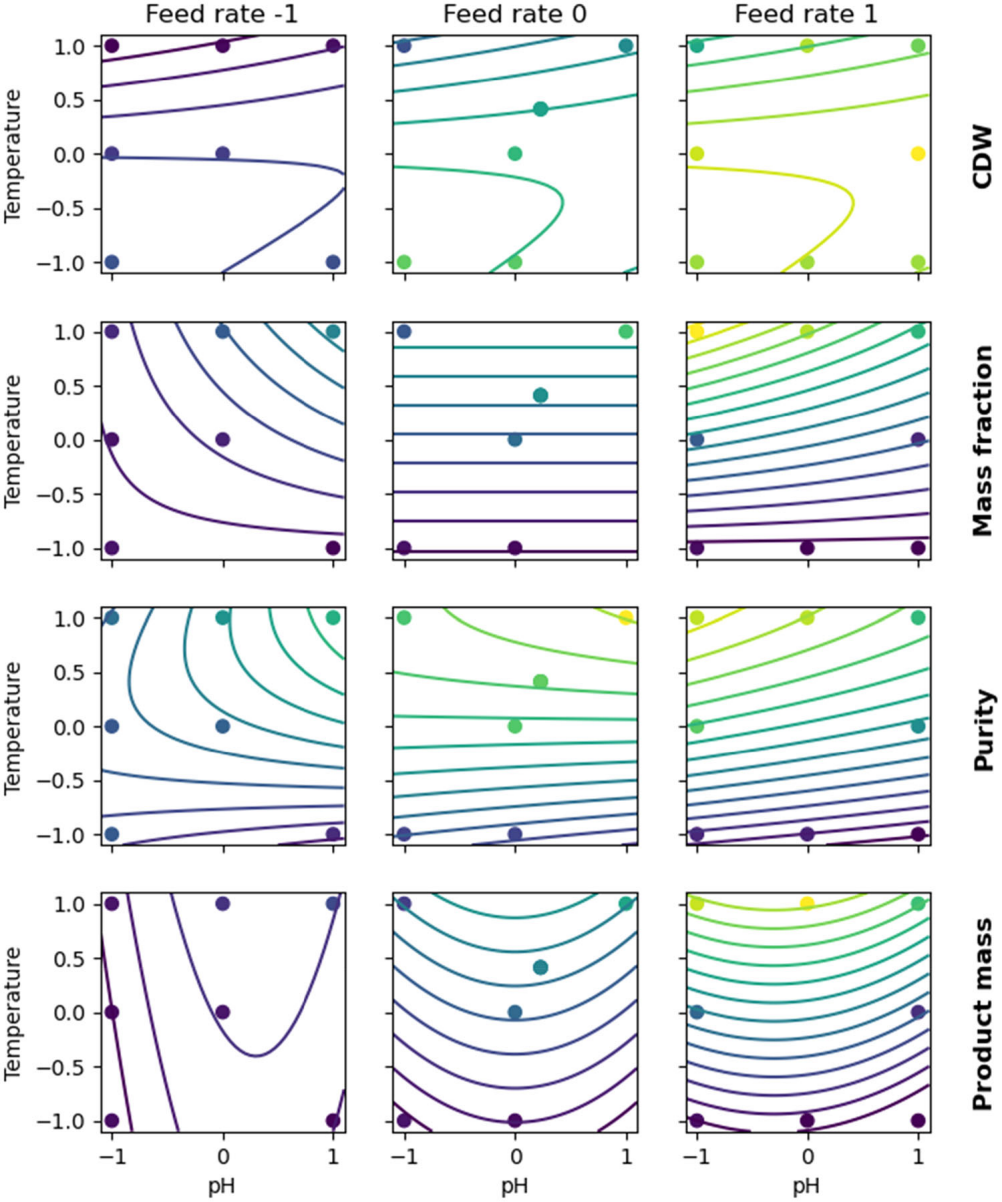
Response surface plots of the process parameters and KPIs including the observations. Each column corresponds to a different feed rate. The used color map represents values continuously from low (dark blue) to high (yellow). Product refers to mass of scFv in IBs determined by CE‐SDS. Row 1: CDW biomass at harvest. Row 2: mass fraction of product per CDW. Row 3: purity after cell lysis determined by CE‐SDS. Row 4: final product mass. To simplify visualization, the cultivation at −0.03 normalized feed rate is omitted from the figure.

Linear quadratic models with interaction terms were calibrated to the KPIs with the normalized process parameters as factors. Quadratic terms were included since an optimum close to the DoE center was expected a priori. The resulting models abstract the relations between process parameters and KPIs confirming the validity of the experimental set. Based on the kernel density estimates (Supporting Information S1: Figure S3), no relevant divergence exists between the two experimental blocks. Furthermore, a high KPI reproducibility was achieved for the triplicate cultivation conditions compared to the overall variability. For the four KPIs, $R^{2}$ values of 0.952, 0.881, 0.944, and 0.891 were reached for the CDW, mass fraction product, purity, and product mass, respectively. The resulting response surfaces are visualized in Figure 2. The model coefficients are listed in Supporting Information S1: Table S4.

The CDW model has the highest coefficient of determination and only includes linear and quadratic terms for the temperature and the feed rate, as well as an interaction term for pH:temperature. In general, higher temperatures lead to increased turnover of the maintenance metabolism (Doran 2012) and reduced substrate flux towards biomass formation. In our experimental setup—same time of product formation phase for all DoE conditions—higher feed rates translate into more total substrate delivered, which explains the higher biomass formation observed. Conversely, the impact of the pH is more confounded when coupled with the temperature. Overall, the observations align well with theoretical considerations (Doran 2012).

The observed response surfaces for both product mass fraction and purity (Figure 2 rows 2 and 3) are highly correlated, with the product purity showing slightly more complex response curvatures. In the most productive cultivation conditions, nearly pure product was obtained. On the other hand, the IB purity severely dropped for low product mass fractions. Feed rate (or total carbon delivered) and temperature had a positive impact for both product mass fraction and purity, while the pH showed more complex nonlinear interaction effects.

Finally, the highest final product mass is observed at pH 6.85, 35°C, and high feed rate. The response surface model predicts even higher total product mass at slightly lower pH values in conjunction with higher feed rates and temperatures. Proceeding in this direction, the host organism will ultimately reach its limit temperature (increased death rate and maintenance) and feed rate (risk of generating overflow metabolism). As product mass is commonly the utmost important process KPI, it would be concluded that the process optimum for this specific protein must exist outside of the experimental space covered in this study with a higher feed rate, a temperature above 35°C and a pH slightly below 6.85.

### 3.2 Analysis of Biophysical Data

One focus of the study was to understand the variations introduced into the lysed pellet due to changes in the cultivation process conditions. The study used analytical methods that detect non‐proteinous contaminants, as well as structural aspects of the protein within the IBs and particle‐size related information. A panel of spectroscopic methods consisting of fluorescence spectroscopy (mainly measuring hydrophobicity around aromatic amino acid residues via their intrinsic fluorescence), FTIR spectroscopy (protein secondary structure, chemical composition), Raman spectroscopy (protein secondary structure, chemical composition, protein tertiary structural aspects), and LD (particle sizing) were selected (Jiskoot and Crommelin 2005; Rolinger et al. 2020; Rüdt et al. 2017).

To improve the analytes' signal compared to the background, water was removed from the samples by lyophilization before the spectroscopic measurements. Lyophilization has been reported to affect the protein structure (Jiskoot and Crommelin 2005). However, in the current study, lyophilization poses a small risk: First, the compositional analysis is not significantly affected since the relevant components (protein, lipids, nucleic acids, cell wall fragments) are non‐volatile. Second, IBs are known for their high stability which counteracts protein structural changes due to the removal of water (Buscajoni et al. 2022; Dürauer et al. 2009; García‐Fruitós et al. 2012; Rinas et al. 2017). Third, even if protein structural changes occur, it will likely be a systematic bias not impacting the statistical interpretation. Finally, the drying of IBs for spectroscopic analysis is an established method in literature, supporting its usage (Kopp and Spadiut 2023).

**Figure 3:**
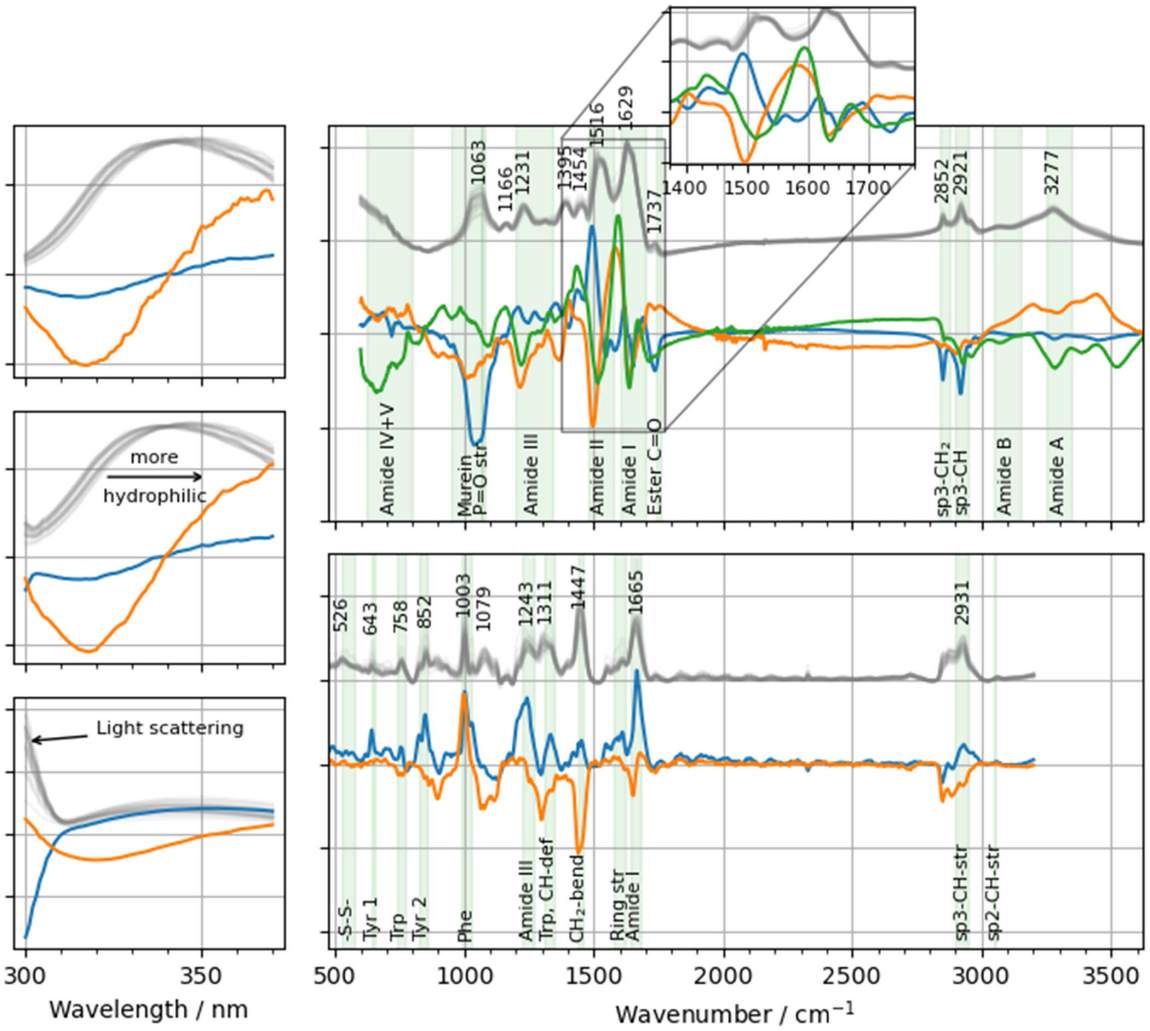
Loadings of the PCAs for the different spectroscopic methods. For the FTIR and Raman loadings, the most important bands are annotated with a chemical group and wavenumber maxima. PC 1, PC 2, and PC 3 are marked in blue, orange, and green, respectively. In gray, the baseline‐subtracted and normalized spectra of the 24 cultivations are shown before mean‐centering. For FTIR and Raman, the spectra were offset from zero to the first horizontal gridline to simplify the reading of the figure. Left column: fluorescence data with an excitation at 260 nm (top), 270 nm (middle), and 280 nm (bottom). Top right: FTIR spectra with the inset zooming onto the range of the amide I and II bands. Bottom right: Raman spectra.

The preprocessed spectra are displayed in Figure 3. The spectra were factorized by PCA to obtain a reduced number of variables summarizing the main variations observed with each spectroscopic method.

The fluorescence spectra (Figure 3 left column) were summarized by two PCs with an explained variance fraction of 89.2% and 9.1%, respectively. The first PC collects information related to emission shifts to longer wavelengths around 340 nm as well as reduced intensities close to 300 nm for excitation at 280 nm. The second PC also collects an emission shift to longer wavelengths but with only minor changes around 300 nm. As the emission around 340 nm is mostly related to tryptophan fluorescence (Lakowicz 2006) such a shift to longer wavelengths implies an overall more hydrophilic environment around tryptophan. With tryptophan typically being buried in proteins and not being exposed to water, a red shift of fluorescence emission compared to the folded protein can be indicative of increased water exposure (i.e., an unfolded state). On the IB level, the changing hydrophobicity is likely caused by a more porous IB structure as well as a reduced quantity of lipids and other highly hydrophobic compounds per product mass (see Supporting Information S1: Figure S4). The recorded intensities between 300 nm and 310 nm for an excitation at 280 nm are mainly related to light scattering due to imperfect excitation emission monochromator performance of the used plate reader (excitation bandwidth 15 nm, emission bandwidth 20 nm as per instrument specifications) and provided an additional information source for observing the biophysical characteristics of the IBs.

The FTIR spectra (Figure 3 top right) show multiple bands, most prominently related to the amide vibrations of the protein backbone with amide I at 1629 cm^−1^, amide II at 1516 cm^−1^, amide III at 1231 cm^−1^ as well as other amide vibrations (Barth 2007; Jiskoot and Crommelin 2005; Schweitzer‐Stenner 2006). While the α‐helix and the unordered amide I bands display a maximum around 1654 cm^−1^ (Barth 2007), the low wavenumber of the amide I band indicates an excess of β‐sheets (Barth 2007) or even amyloid structural elements (Moran and Zanni 2014) which is typical for the aggregated state of IBs (Rinas et al. 2017). The shoulder at 1650 cm^‐1^ indicates that a minority of the secondary structure of the IB remains most likely in a disordered structure (Barth 2007) since the native product does not contain any α‐helix as per *in‐silico* structure prediction (Supporting Information S1: Figure S5) (Jumper et al. 2021). Other relevant bands in the spectra are the C─H stretching vibrations at 2852 cm^−1^ and 2921 cm^−1^ and the ester vibration at 1737 cm^−1^, attributable to lipids (Harz et al. 2009). Finally, the broad band at 1063 cm^−1^ is attributable to the C─C, C─O, and O─H vibrations of the murein in bacterial cell walls (Jiang et al. 2004). P═O vibrations may also contribute to this region. It is assumed that the band at 1166 cm^−1^ is caused by organic phosphate components (e.g., in phospholipids or DNA) (Jiang et al. 2004).

Three PCs were retained during PCA covering 55.7%, 24.8%, and 9.1% of the total variation. PC 1 seems to be mainly related to the fraction of cell wall and membrane fragments in the samples and thus higher scores indicate less cell debris. Interestingly, the amide I, II, and III bands counterintuitively did not reciprocally increase with decreasing cell debris (bands at 1063, 1737, 2852, and 2921 cm^−1^). This is likely due to the normalization scheme taking the strong protein features as reference. Additionally, PC 1 shows high loadings around 1490 cm^−1^, indicating a band shift of amide II towards lower wavenumbers (Barth 2007), presumably due to the lower abundance of cell wall associated peptides.

FTIR PC 2 and 3 collect mainly variations in the region of the amide I, II, and, to a lesser degree, III bands. Positive loadings indicate a further shift of the amide I band to lower wavenumbers indicating a stronger aggregation and stronger alignment in intramolecular β‐sheets (Barth 2007; Moran and Zanni 2014). Amyloid fibers generate delocalized vibrations which results in a peak shift to wavenumbers below 1630 cm^−1^ in FTIR spectra with some absorption below 1600 cm^−1^ for certain amyloid structures (Moran and Zanni 2014; Ostapchenko et al. 2010). Secondary structural changes also affect the amide II and III bands. It is therefore expected that PC 2 and 3 also collect variation in these regions. Further considerations on the loadings of FTIR PC 2 and 3 are given in Supplementary Section 2.2.

The Raman spectra (Figure 3 bottom right) are largely dominated by protein bands. The spectra feature all relevant bands associated with amide vibrations, vibrations of the aromatic amino acids, and disulfide bridges (Jiskoot and Crommelin 2005; Rygula et al. 2013). One notable band not associated with protein vibrations occurs at 1079 cm^−1^. As with the FTIR spectra, this band is likely related to the murein carbohydrate vibrations (Harz et al. 2009). Interestingly, while a distinct peak at 526 cm^−1^ caused by disulfide vibrations can be seen, no similar pattern is visible for S‐H vibrations around 2550 cm^−1^ indicating that most cysteines are in their oxidized form. Thus, prevalent disulfide bridges may lead to intramolecular cross‐linking limiting the solubilization of IBs without a reducing agent. The regular pattern above 1800 cm^−1^ is most likely due to background fluorescence or Rayleigh scattering effects in combination with the oscillating efficiency of the optical setup. This pattern has been discussed in more detail elsewhere (Goldrick et al. 2020; Whelan et al. 2012).

Raman spectra were decomposed into two PCs, collecting 52.5% and 24.5% of the total variation. PC 1 of the Raman data collects variations in the protein band intensities. Higher scores lead to an increase in the protein‐related bands and therefore indicate a higher fraction of protein. PC 2 is affected by bands related to amide bonds and CH‐groups (cf. overlap between loadings and marked protein bands in Figure 3). It therefore seems to quantify cell debris contribution with higher scores indicating less cell debris. PC 2 also receives a weak contribution from amide I vibration, which may potentially be related to the amide I of the murein cross‐linking oligopeptides (Jiang et al. 2004).

LD yielded particle size distributions post cell lysis. Since the IBs were not purified before analysis, the resulting particle distributions include not only the distribution of IB sizes but also incorporate cell debris and agglomerates. Large particles ( > 5 µm) were removed by filtration. Filtration is supported by literature not finding any IBs in a similar size range (Peternel et al. 2008; Wurm et al. 2018). In addition, it has already been observed that the bacterial cell walls hardly change with cultivation conditions and the investigated pH range (Jiang et al. 2004). Consequently, cell wall fragments were assumed to affect the IB size distribution measurements consistently. Particle sizes were verified with orthogonal dynamic light scattering measurements which resulted in very similar sizes (data not shown).

**Figure 4:**
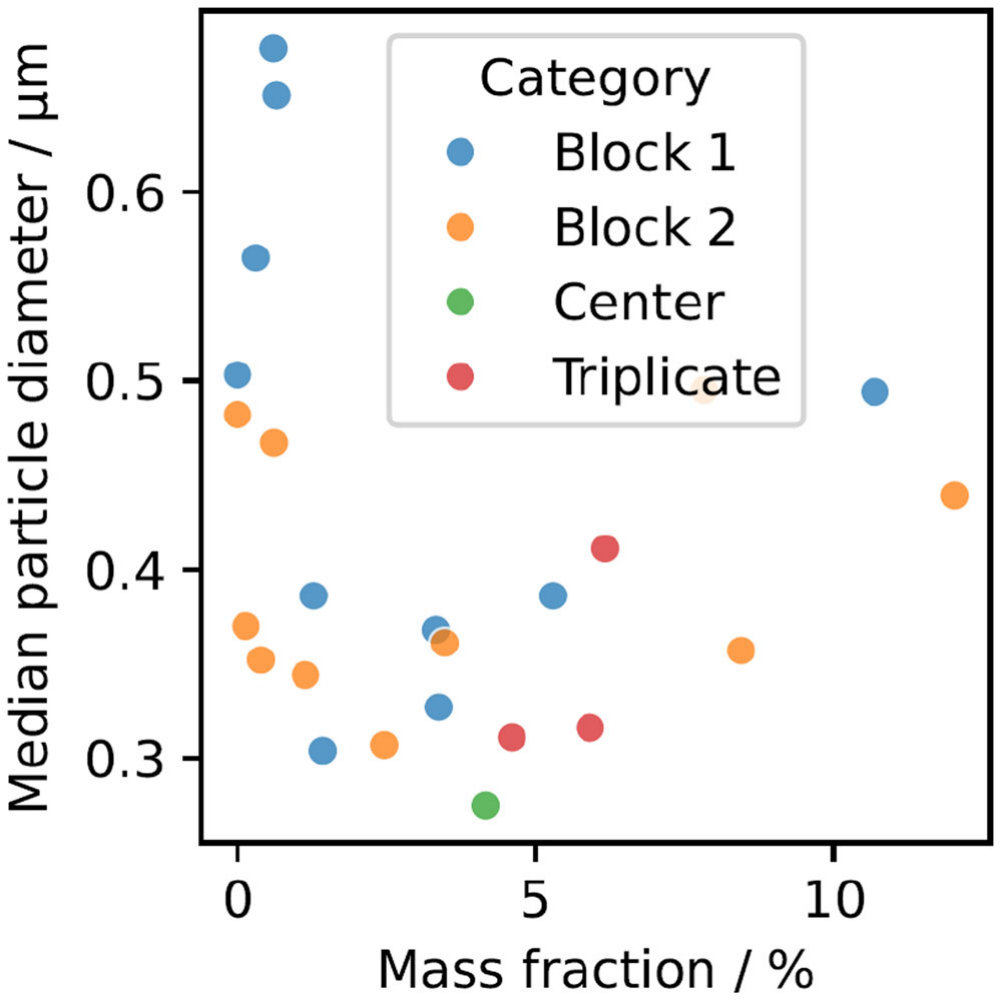
Median particle diameter as a function of the mass fraction of product per CDW. The data is colored by either originating from cultivation block 1 (blue) or 2 (orange). The triplicate operating conditions are marked in red and the center of the design space in green.

Plotting the median particle diameter against the mass fraction of the product reveals a clear dependency (Figure 4): The largest particles are observed at very low product mass fractions, where also purity is low. A minimum particle size is reached for intermediate product mass fractions with a slow increase for larger mass fractions. At the lowest IB mass fractions, the particle distribution is likely biased by intact cells or agglomerated IBs and cell debris resulting in large observed particle diameters (Reichelt et al. 2017; Wong et al. 1997) although remaining significantly smaller than some agglomerates reported in literature (Walther et al. 2013). In the intermediate case, the smaller mass fraction of cell debris apparently reduces the agglomeration propensity. At high productivities, more product is stored in IBs during cultivation leading to bigger particles (Slouka et al. 2018; Slouka et al. 2019).

### 3.3 Effect of Cultivation Parameters on Biophysical Characteristics of IBs

It was investigated if the biophysical attributes of the IBs could be described by the variation in cultivation parameters. For all biophysical attributes, the data contained statistical evidence that cultivation parameters were relevant. Model selection never favored a naïve, constant model. The effect of cultivation conditions on IB biophysical attributes is also strongly supported by literature (García‐Fruitós et al. 2012; Jevševar et al. 2005; Margreiter et al. 2008; Reichelt et al. 2017; Slouka et al. 2018). A summary table of all models with coefficients of determination and the regression coefficients is given in Supporting Information S1: Table S5.

While the cultivation parameters were found to consistently affect the biophysical attributes of the IBs, the observed model quality for correlating the cultivation parameters to the biophysical attributes ranged from good over reasonable to poor. Good and reasonable models were found for the median particle diameter, fluorescence score 1, and Raman score 1 with coefficients of determination of 0.795, 0.690, and 0.531, respectively. Models of mediocre quality were observed for fluorescence score 2, FTIR score 2, and FTIR score 1 with coefficients of determination of 0.438, 0.425, and 0.384, respectively. Poor model quality was also observed for the Raman score 2 and the FTIR score 3 with coefficients of determination of 0.236 and 0.124, respectively.

**Figure 5:**
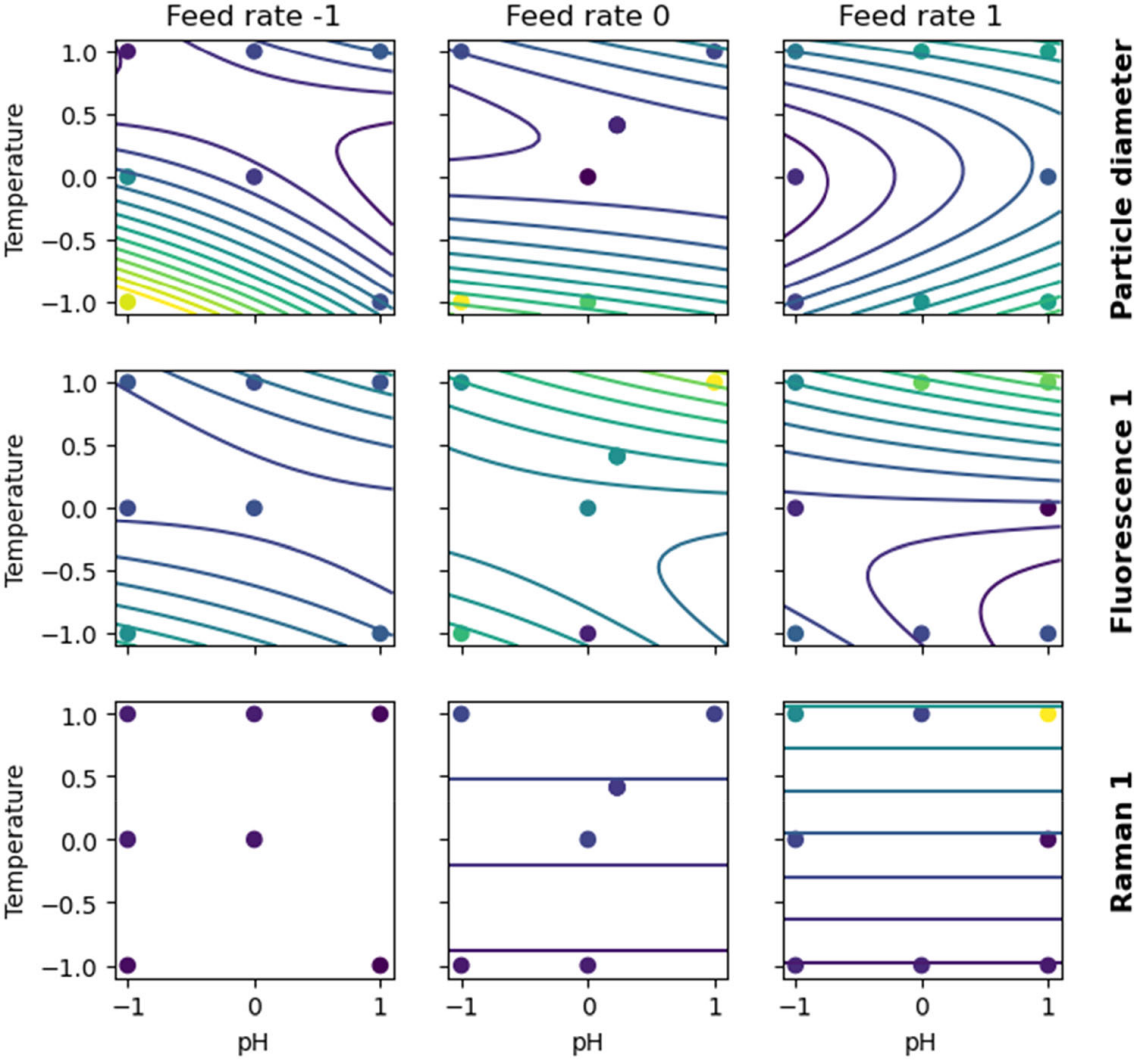
Response surface plots of the process parameters and the biophysical attributes including the observations. Each column corresponds to a different feed rate, and each row to a different biophysical attribute as response variables. To simplify visualization, the cultivation at a normalized feed rate of −0.03 was omitted from the figure. The used color map represents values continuously from low (dark blue) to high (yellow).

Only models with a coefficient of determination > 0.5 (i.e. good and reasonable models) are discussed in more detail. Response surface plots of those models are shown in Figure 5. Models with a coefficient of determination ≤ 0.5 explain less than half of the observed variability. These models are not discussed in more detail since they are either strongly affected by uncontrolled factors or demonstrate strongly a nonlinear behavior which could not be captured with the used linear‐quadratic model. Remarkably, models for FTIR scores 2 and 3 are below this cutoff, meaning that the amyloid content in IBs can only inadequately be explained by the process parameters. This is interesting since the amyloid content has previously been described as important for the refolding behavior (Jevševar et al. 2005). It furthermore means that the control of the amyloid content is difficult for the investigated scFv.

The largest IBs were found at a low pH, low temperature and low to medium feed rates. However, the relationship between cultivation conditions and IB size is nonlinear, and particles of intermediate size were found with high pH values and feed rates combined, for both high and low temperatures. Furthermore, literature reports that the IB size is correlated to the IB purity (Wurm et al. 2018) which is tentatively supported by the data shown in this study (cf. Figures 2 and  5). Discrepancies exist for low purities, where the largest particles were observed in this study. However, these discrepancies might be explained by the different analytical methods (CGE‐SDS and LD in this study; denatured SEC and scanning electron microscopy by Wurm et al. [2018]), with LD being faster but not providing an easy detection of agglomerates or foreign particles compared to scanning electron microscopy (Reichelt et al. 2017).

High fluorescence scores 1 indicate a hydrophilic behavior around tryptophan coupled with low light scattering effects. Conditions of high temperature and high pH seem to result in the highest fluorescence score 1 values. Fluorescence score 1 follows a similar profile to particles sizes, i.e. if the particle diameters are high, fluorescence score 1 is high and vice versa. Larger particles have a lower surface to volume ratio, which leads to less tryptophan residues exposed on the particle surface. The internal residues are less accessible for hydrophobic contaminants (e.g., cell membrane residues) and could therefore contribute to more hydrophilic fluorescence spectra.

The Raman scores 1 mainly capture protein bands and are therefore indicative of protein mass fractions in the freeze‐dried cell lysis pellets. Conditions of high feed rates and high temperature show the highest product mass fractions as well as the highest Raman score 1. While Raman scores 1 noticeably deviate from the measured product mass fraction, the corresponding model still captures the general trend.

In summary, by setting all cultivation parameters simultaneously either to their high or to their low levels, the particles become larger and more hydrophilic. By simultaneously moving all cultivation parameters to their high levels, the highest protein content is measured by Raman spectroscopy. The best cultivation conditions from a biophysical point of view are therefore given, when all cultivation parameters are high.

### 3.4 Effects of IB Pellet Biophysical Characteristics on Refolding

In the last stage of the study, the biophysical characteristics were linked to the solubilization and refolding process. The prediction of two KPIs was assessed: First, it was evaluated whether the biophysical attributes could be used to predict the protein mass fraction in the washed wet IB pellet. Second, the effect of the biophysical properties on the refolding yields was assessed.

**Figure 6:**
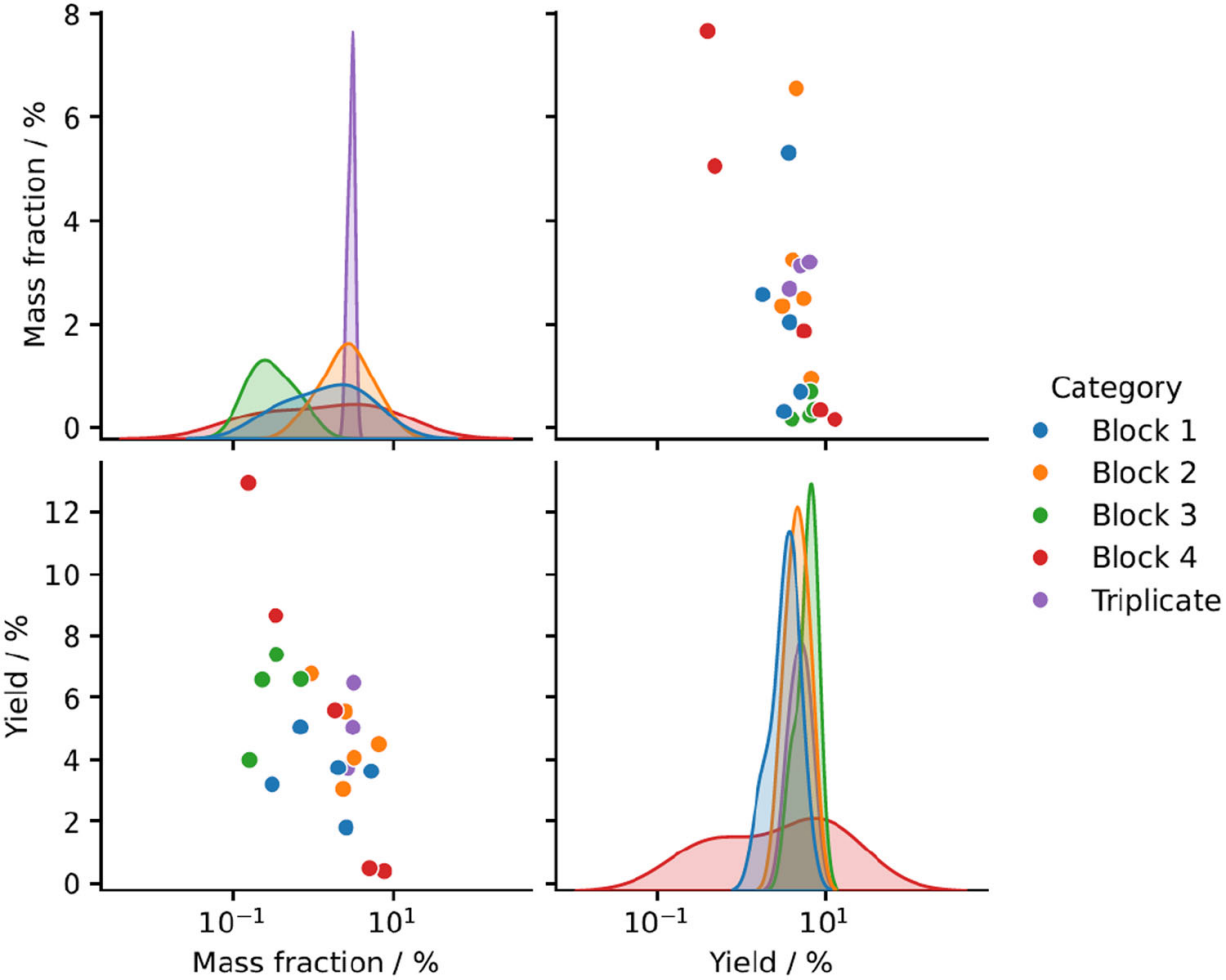
Pair plot of different KPIs for solubilization and refolding. The *x*‐axes are logarithmic. Blocks 1–4 refer to the four experimental blocks that the samples were split into for IB solubilization and refolding. The triplicate operating conditions are marked in purple.

Figure 6 shows a pair plot of the two KPIs. Interestingly, a negative correlation between product mass fraction in washed wet pellet and refolding yield is visible. Thus, the higher the product concentration in the washed wet pellet, the lower the yield during refolding. While this connection might seem surprising, the effect is likely caused by controlling the concentration of wet pellet instead of the product concentration during solubilization and refolding. Lower product content in the washed wet pellet leads to lower product concentrations during refolding, therefore a lower propensity of aggregation, and a higher refolding yield, a behavior which is well known from literature (Buscajoni et al. 2022; Eiberle and Jungbauer 2010; Vallejo and Rinas 2004). The connection highlights how a hidden coupling may be introduced from cultivation to refolding by choosing a suboptimal control strategy for the production process. As an improved control strategy, it might be useful to introduce an in‐process control with a feedback loop to adjust the protein concentration after solubilization.

Based on the biophysical attributes, models were calibrated for predicting the two refolding KPIs. In contrast to all previous models, the model type was switched from OLS to GLM with a logarithmic linker function and normal errors. The reason for this approach was to accommodate the large fraction of predictions close to null. OLS models were evaluated as well, however, based on the prominence of the best GLM model, the OLS models had almost no experimental support (ΔAICc≥7.6 and ΔAICc≥9.2 for the mass fraction and yield, respectively) and were therefore not further considered.

**Figure 7:**
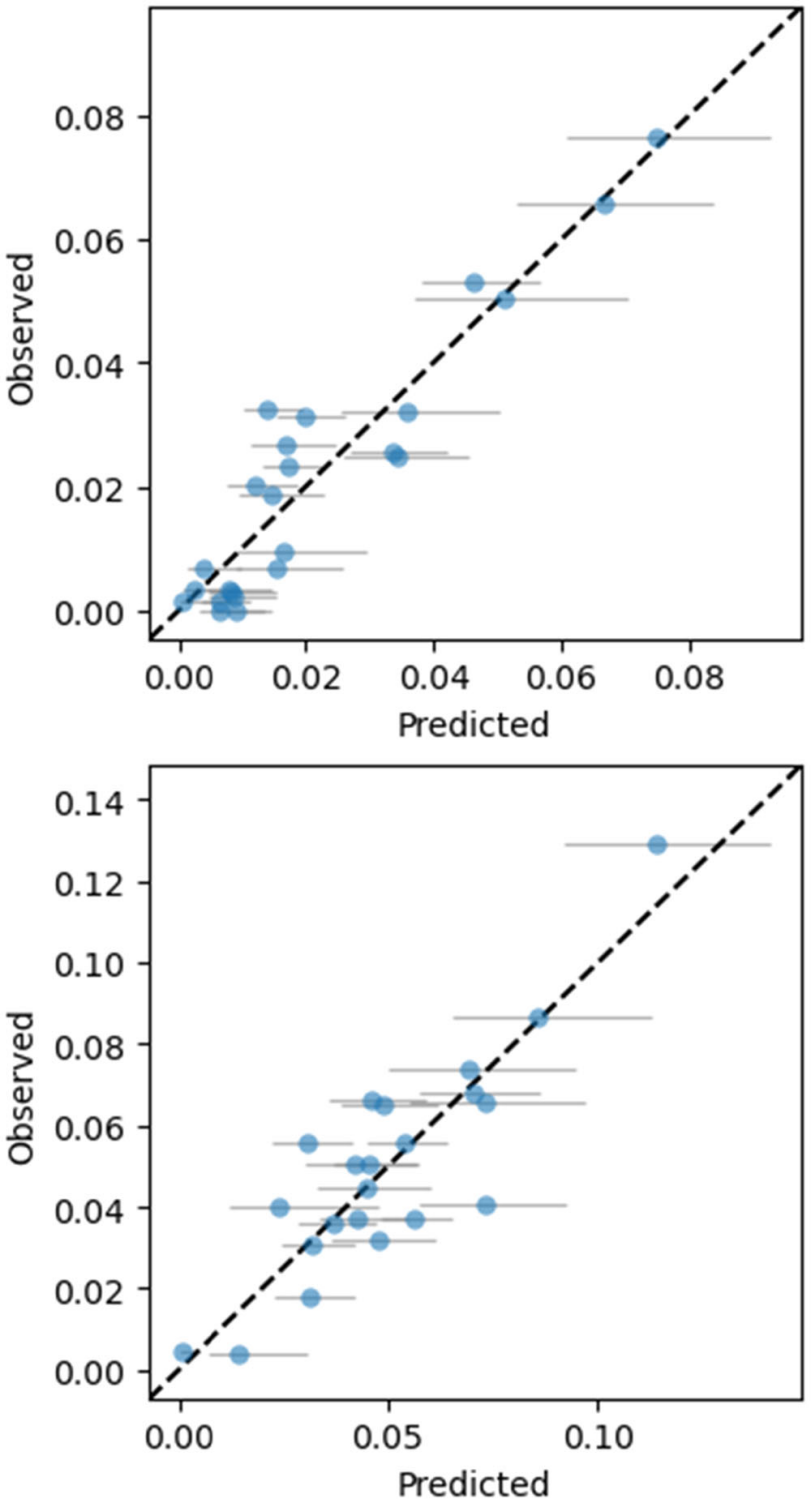
Prediction quality of different KPIs for solubilization and refolding from biophysical attributes. The gray bars indicate the 95% confidence intervals of the model predictions. Top: product mass in washed cell pellet. Bottom: yield over the whole refolding process.

Table 2 summarizes the model quality metrics as well as the regression coefficients of the selected GLM models. The models reach a high prediction quality during calibration as shown by their good pseudo R^2^ values. Furthermore, they perform robustly during cross‐validation with a limited increase in the cross‐validation errors compared to the calibration errors. A parity plot of the model predictions for both responses is shown in Figure 7.

**Table 2:** Summary of the linear regression models which use the biophysical attributes as factors and either the mass fraction product per wet pellet or the refolding yield as the responses. The relative RMSECV is normalized by the maximum of each response. The lower half of the table shows the relevant regression coefficients. RMSEC: Root mean squared error of calibration, RMSECV: Root mean squared error of cross‐validation.

Parameter	Mass fraction product	Refolding yield
Pseudo *R* ^2^	0.996	0.906
Model prominence Δ AICc	0.92	1.55
RMSEC/%	0.733	1.33
RMSECV/%	0.921	1.99
Relative RMSECV/%	11.8	15.4
Intercept	−4.2821	−3.2065
Fluorescence score 2	0.3836	0
FTIR score 2	−0.6308	−0.3277
FTIR score 3	−0.5957	−0.2136
Raman score 1	0.6479	−0.8976
Raman score 2	0.4748	0
Median particle diameter	0	0.2188

With respect to the model for the mass fraction of product, three biophysical attributes contribute positively (Fluorescence score 2, Raman score 1 and 2) while two biophysical attributes contribute negatively (FTIR score 1 and 2) to the product mass fraction:

The positive contribution of Raman scores 1 and 2 is expected since the two PCs are related to the protein signal in the lysed cell pellet. The higher the scores, the higher the protein content. Fluorescence score 2 summarizes the hydrophilicity around tryptophan without light scattering contributions. Higher scores indicate a more hydrophilic environment. This was interpreted as a metric for the ratio of IB to hydrophobic lipids from the cell membrane. Consequently, a more hydrophilic environment indicates a higher protein‐to‐total mass ratio.

Higher FTIR scores 2 and 3 predict a lower measured mass fraction of product in the cell lysis pellet. As discussed in Section 3.2, PC 2 and 3 are linked to the occurrence of large amyloid structures in the IB. In literature, it has already been reported that the amyloid structures are especially robust against solubilization (Rinas et al. 2017; Upadhyay et al. 2012). The reported amyloid structures here are remarkable due to their low vibrational frequencies interpreted as especially large and densely coupled amyloids. It can therefore be hypothesized that a fraction of the amyloid structures cannot be solubilized with the harsh conditions used during the analytical procedure (Dürauer et al. 2009; Walther et al. 2023; Walther et al. 2022), resulting in a negative impact of these amyloid structures on the measured product mass fraction. From an analytical perspective, this may be a limitation of the currently used method which needs to be considered in future method development. A special focus must be set towards validating that the solubilization conditions are adequate independent of the amyloid content of the IBs.

The refolding yield model includes four biophysical attributes with one impacting the yield positively (median particle diameter) and three negatively (Raman score 1, FTIR score 1 and 2):

The median particle diameter has a positive impact on the refolding yield. This is consistent with the hypothesis stated in the literature that larger IB sizes are beneficial for the process since they minimize the surface‐to‐volume ratio limiting the adsorption of contaminants (Slouka et al. 2018; Wurm et al. 2018). This study presents for the first time evidence over a whole process that the refolding yield is positively affected by larger IBs by experimentally linking cultivation over biophysical attributes to refolding. Large IBs are observed at high cultivation titers. Therefore, the standard upstream process development objective of maximizing the product titer positively impacts the refolding performance.

Studies by Walther et al. (2013) and Walther et al. (2023) indicate that large agglomerates of IBs negatively affect solubilization. In our study, we did not monitor the size of IB agglomerates, as they were filtered out before LD measurements. However, the high explained variance of our model suggests that any variability due to size differences in IB agglomerates is small for this product and process.

The refolding yield is furthermore negatively affected by Raman score 1. As discussed in the previous section, Raman score 1 is correlated to the protein mass fraction in the cell lysis pellet. Due to the control strategy, a higher Raman score 1 is, therefore, indicative of a higher product concentration during the refolding process. The higher the product concentration, the bigger is however also the probability of intermolecular interactions and aggregation, therefore lowering the refolding yield (Buscajoni et al. 2022; Eiberle and Jungbauer 2010; Vallejo and Rinas 2004) (see also Figure 6).

Finally, the FTIR score 1 and 2 affect the refolding yield negatively. The reason for this behavior is presumably the same as for the negative impact of the two scores on the product mass fraction: the occurrence of stable amyloid structures interferes with the solubilization (Jevševar et al. 2005; Singh et al. 2015; Walther et al. 2023; Walther et al. 2022). Since the solubilization conditions in the process are milder than in the analytical procedure (room temperature and no SDS), the solubilization is thought to be even less effective. The incomplete solubilization in turn results in a lower observed refolding yield.

Based on the collected evidence, several measures appear beneficial to enhance process understanding and to control the effects of upstream processing on IB refolding. These measures include the measurement and adjustment of product concentration in routine manufacturing after solubilization to ensure tight concentration control during refolding. Additionally, the formation of amyloid structures, detectable by FTIR spectroscopy, should be minimized during process development. Finally, IB sizes should be maximized, which could be easily monitored with LD, for example. Together, these approaches can contribute to a more robust and controlled refolding process.

## 4 Conclusion

In biopharmaceutical production, it is frequently hypothesized that cultivation conditions of IB processes affect process performance, not only at a cultivation level, but also during the subsequent refolding. Since the exact mode of action remains generally disputed, the hypothesis is often rejected based on theoretical considerations. In this study, anti‐desipramine scFv, expressed in *E. coli* fed‐batch cultivations and subjected to dilution‐refolding, was used as an industrially relevant model process to investigate this hypothesis in a designed study. By systematically varying the cultivation conditions via a DoE approach, the study gained insights into the complex relationships between cultivation and refolding by investigating the biophysical properties of the IBs as a link.

For the model system, higher feed rates resulted in increased cell density and product mass. Meanwhile, nonlinear influences of temperature, pH value, and interaction terms on cultivation performance were observed. For example, elevated temperatures raised product mass and purity at high feed rates, whereas this correlation was less pronounced for slow feeding. Also, a clear dependency between cultivation parameters and refolding performance could be observed where elevated product mass fractions obtained during cultivation influenced refolding performance negatively due to hidden coupling of the mass fraction to the concentration during refolding. To decouple cultivation from refolding, a product concentration adjustment after solubilization may be routinely implemented in the production control strategy.

The IB biophysical properties were characterized to gain insights into impurity levels, hydrophobicity, particle size, and secondary structural elements (such as β‐sheets, amyloid formation, and unstructured regions). Larger IBs, observed with all cultivation parameters simultaneously set to either low or high levels, easily monitored by for example, LD, enhanced protein refolding efficiency. In contrast, an increase of amyloid structures, accessible through for example, FTIR spectroscopy, resulted in lower refolding yields explained by incomplete IB solubilization. Notably, the amyloid content could not be strongly linked to the cultivation process parameters, showing that the secondary structure of IBs is affected either in a strongly nonlinear manner by the varied process parameters or depends on uncontrolled factors. Either way, this dependence should be further considered during process development to minimize the impact of cultivation on refolding.

With the IB biophysical properties establishing a link between cultivation and refolding performance, the importance of an integrated process development strategy emerges, where the impact of upstream on downstream must be considered. The methodology presented in this study supports such an integrated process development. While the relationships identified here depend on the specific target protein, host, and process, the methodology is generalizable and should be considered as a template for process development.

## Author Contributions

Matthias Rüdt and Holger Morschett obtained the funding. Matthias Rüdt, Holger Morschett, Aymerick Bussien, Joan Cortada‐Garcia, and Karlheinz Flicker conceived the idea and designed the experiments. Joan Cortada‐Garcia and Hauke Holm performed the cultivation experiments. Aymerick Bussien adapted and executed the cell lysis, IB recovery and refolding, and the analytical and biophysical measurements. Aymerick Bussien and Chiara Mazzucchelli developed and applied the protein L analytical method. Matthias Rüdt performed the data analysis. Matthias Rüdt, Holger Morschett and Joan Cortada‐Garcia interpreted the results. Matthias Rüdt, Aymerick Bussien, Joan Cortada‐Garcia, and Holger Morschett wrote the manuscript and drew the figures. All authors reviewed the manuscript.

## Ethics Statement

The authors declare no conflict of interests and adhered to the common ethical standards when preparing the manuscripts.

## Conflicts of Interest

The authors declare no conflicts of interest.

## Supporting information


**Supplementary Figure 1:** Schematic representation of the cultivation protocol with the different phases of the experiments and key events. **Supplementary Figure 2:** Workflow of IB recovery, protein solubilization, and refolding, including sampling points and analytics. **Supplementary Figure 3:** Pair plot of different key performance indicators of the cultivation process. **Supplementary Figure 4:** Scatter plot of FTIR score 1 with the two fluorescence scores. **Supplementary Figure 5:** Structure of anti‐desipramine antibody single chain fragment variable in‐silico predicted using AlphaFold version 2 (Jumper et al., 2021). **Supplementary Table 1:** Composition of buffers utilized for protL HPLC. **Supplementary Table 2:** Scoring of evaluated process parameters. Based on own data and literature, individual parameters were scored 1‐10 regarding “likelihood of parameter to have an influence”, “expected intensity of influence” and “controllability of the parameter”. The overall score was calculated as the product of the individual scores and the top three ranks (except for induction strength) were considered for the D‐Optimal design. **Supplementary Table 3:** D‐Optimal design including blocking for cultivation and refolding. **Supplementary Table 4:** Summary of OLS models correlating process parameters to KPIs. Empty cells correspond to a zero coefficient. **Supplementary Table 5:** Summary of OLS models correlating process parameters to biophysical attributes. Empty cells correspond to a zero coefficient.

## Data Availability

Data is available as an Open Research Data set.

## References

[bit70033-bib-0001] de Aguiar, P. F. , B. Bourguignon , M. S. Khots , D. L. Massart , and R. Phan‐Than‐Luu . 1995. “D‐Optimal Designs.” Chemometrics and Intelligent Laboratory Systems 30, no. 2: 199–210. 10.1016/0169-7439(94)00076-X.

[bit70033-bib-0002] Barth, A. 2007. “Infrared Spectroscopy of Proteins.” Biochimica et Biophysica Acta (BBA) ‐ Bioenergetics 1767, no. 9: 1073–1101. 10.1016/j.bbabio.2007.06.004. 17692815

[bit70033-bib-0003] Bhatwa, A. , W. Wang , Y. I. Hassan , N. Abraham , X.‐Z. Li , and T. Zhou . 2021. “Challenges Associated With the Formation of Recombinant Protein Inclusion Bodies in *Escherichia coli* and Strategies to Address Them for Industrial Applications.” Frontiers in Bioengineering and Biotechnology 9: 630551. 10.3389/fbioe.2021.630551. 33644021 PMC7902521

[bit70033-bib-0004] Burnham, K. P. , and D. R. Anderson . 2002. Model Selection and Multimodel Inference: A Practical Information ‐ Theoretic Approach. Springer. 10.1007/b97636.

[bit70033-bib-0005] Buscajoni, L. , M. C. Martinetz , M. Berkemeyer , and C. Brocard . 2022. “Refolding in the Modern Biopharmaceutical Industry.” Biotechnology Advances 61: 108050. 10.1016/j.biotechadv.2022.108050. 36252795

[bit70033-bib-0006] Castellanos‐Mendoza, A. , R. M. Castro‐Acosta , A. Olvera , et al. 2014. “Influence of Ph Control in the Formation of Inclusion Bodies During Production of Recombinant Sphingomyelinase‐D in *Escherichia coli* .” Microbial Cell Factories 13, no. 1: 137. 10.1186/s12934-014-0137-9. 25213001 PMC4177172

[bit70033-bib-0007] Cox, D. R. , and E. J. Snell . 1989. Analysis of Binary Data, 2nd ed. Routledge. 10.1201/9781315137391.

[bit70033-bib-0008] Demain, A. L. , and P. Vaishnav . 2009. “Production of Recombinant Proteins by Microbes and Higher Organisms.” Biotechnology Advances 27, no. 3: 297–306. 10.1016/j.biotechadv.2009.01.008. 19500547

[bit70033-bib-0009] Doran, P. M. 2012. Bioprocess Engineering Principles, 2nd ed. Elsevier. https://educate.elsevier.com/book/details/9780122208515.

[bit70033-bib-0010] Dürauer, A. , S. Mayer , W. Sprinzl , A. Jungbauer , and R. Hahn . 2009. “High‐Throughput System for Determining Dissolution Kinetics of Inclusion Bodies.” Biotechnology Journal 4, no. 5: 722–729. 10.1002/biot.200800290. 19288514

[bit70033-bib-0011] Ebner, J. , D. Humer , R. Klausser , et al. 2021. “At‐Line Reversed Phase Liquid Chromatography for In‐Process Monitoring of Inclusion Body Solubilization.” Bioengineering 8, no. 6: 78. 10.3390/bioengineering8060078. 34200471 PMC8228044

[bit70033-bib-0012] Eiberle, M. K. , and A. Jungbauer . 2010. “Technical Refolding of Proteins: Do We Have Freedom to Operate?” Biotechnology Journal 5, no. 6: 547–559. 10.1002/biot.201000001. 20518058

[bit70033-bib-0013] Elia, F. , F. Cantini , F. Chiti , C. M. Dobson , and F. Bemporad . 2017. “Direct Conversion of an Enzyme From Native‐Like to Amyloid‐Like Aggregates Within Inclusion Bodies.” Biophysical Journal 112, no. 12: 2540–2551. 10.1016/j.bpj.2017.05.011. 28636911 PMC5479110

[bit70033-bib-0014] Eriksson, L. , E. Johansson , N. Kettaneh‐Wold , C. Wikström , and S. Wold . 2008. Design of Experiments: Principles and Applications, 3rd ed. Umetrics Academy. 10.1002/cem.686.

[bit70033-bib-0015] Fahnert, B. , H. Lilie , and P. Neubauer . 2004. “Inclusion Bodies: Formation and Utilisation.” In Physiological Stress Responses in Bioprocesses, 93–142. Springer Berlin Heidelberg. 10.1007/b93995. 15217157

[bit70033-bib-0016] Freydell, E. J. , M. Ottens , M. Eppink , G. van Dedem , and L. van der Wielen . 2007. “Efficient Solubilization of Inclusion Bodies.” Biotechnology Journal 2, no. 6: 678–684. 10.1002/biot.200700046. 17492713

[bit70033-bib-0017] García‐Fruitós, E. , E. Vázquez , C. Díez‐Gil , et al. 2012. “Bacterial Inclusion Bodies: Making Gold From Waste.” Trends in Biotechnology 30, no. 2: 65–70. 10.1016/j.tibtech.2011.09.003. 22037492

[bit70033-bib-0018] Gasteiger, E. , C. Hoogland , A. Gattiker , et al. 2005. “Protein Identification and Analysis Tools on the ExPASy Server.” In The Proteomics Protocols Handbook, edited by J. M. Walker , 571–607. Humana Press. 10.1385/1-59259-890-0:571.

[bit70033-bib-0019] Goldrick, S. , A. Umprecht , A. Tang , et al. 2020. “High‐Throughput Raman Spectroscopy Combined With Innovate Data Analysis Workflow to Enhance Biopharmaceutical Process Development.” Processes 8, no. 9: 1179. 10.3390/pr8091179.

[bit70033-bib-0020] Gutiérrez‐González, M. , C. Farías , S. Tello , et al. 2019. “Optimization of Culture Conditions for the Expression of Three Different Insoluble Proteins in *Escherichia coli* .” Scientific Reports 9, no. 1: 16850. 10.1038/s41598-019-53200-7. 31727948 PMC6856375

[bit70033-bib-0021] Harris, C. R. , K. J. Millman , S. J. van der Walt , et al. 2020. “Array Programming With Numpy.” Nature 585, no. 7825: 357–362. 10.1038/s41586-020-2649-2. 32939066 PMC7759461

[bit70033-bib-0022] Harz, M. , P. Rösch , and J. Popp . 2009. “Vibrational Spectroscopy ‐ a Powerful Tool for the Rapid Identification of Microbial Cells At the Single‐Cell Level.” Cytometry Part A 75A, no. 2: 104–113. 10.1002/cyto.a.20682. 19156822

[bit70033-bib-0023] Hernandez, A. 2013. “Effect of Culture Conditions and Signal Peptide on Production of Human Recombinant N‐acetylgalactosamine‐6‐sulfate Sulfatase In *Escherichia coli* BL21.” Journal of Microbiology and Biotechnology 23, no. 5: 689–698. 10.4014/jmb.1211.11044. 23648860

[bit70033-bib-0024] Hunter, J. D. 2007. “Matplotlib: A 2D Graphics Environment.” Computing in Science & Engineering 9, no. 3: 90–95. 10.1109/MCSE.2007.55.

[bit70033-bib-0025] Jevševar, S. , V. Gaberc‐Porekar , I. Fonda , B. Podobnik , J. Grdadolnik , and V. Menart . 2005. “Production of Nonclassical Inclusion Bodies From Which Correctly Folded Protein Can Be Extracted.” Biotechnology Progress 21, no. 2: 632–639. 10.1021/bp0497839. 15801811

[bit70033-bib-0026] Jiang, W. , A. Saxena , B. Song , B. B. Ward , T. J. Beveridge , and S. C. B. Myneni . 2004. “Elucidation of Functional Groups on Gram‐Positive and Gram‐Negative Bacterial Surfaces Using Infrared Spectroscopy.” Langmuir 20, no. 26: 11433–11442. 10.1021/la049043+. 15595767

[bit70033-bib-0027] Jiskoot, W. , and D. Crommelin . 2005. Methods for Structural Analysis of Protein Pharmaceuticals. Springer Science & Business Media. https://books.google.ch/books?id=CyeilBm5az8C.

[bit70033-bib-0028] Jumper, J. , R. Evans , A. Pritzel , et al. 2021. “Highly Accurate Protein Structure Prediction With AlphaFold.” Nature 596, no. 7873: 583–589. 10.1038/s41586-021-03819-2. 34265844 PMC8371605

[bit70033-bib-0029] Kateja, N. , H. Agarwal , V. Hebbi , and A. S. Rathore . 2017. “Integrated Continuous Processing of Proteins Expressed as Inclusion Bodies: GCSF as a Case Study.” Biotechnology Progress 33, no. 4: 998–1009. 10.1002/btpr.2413. 27977908

[bit70033-bib-0030] Kluyver, T. , B. Ragan‐Kelley , F. Pérez , et al. 2016. Jupyter Notebooks ‐ a Publishing Format for Reproducible Computational Workflows. 20th International Conference on Electronic Publishing.

[bit70033-bib-0031] Kopp, J. , and O. Spadiut . 2023. Inclusion Bodies: Methods and Protocols, 1st ed. Humana. 10.1007/978-1-0716-2930-7. 36656513

[bit70033-bib-0032] Lakowicz, J. R. 2006. Principles of Fluorescence Spectroscopy. Springer. 10.1007/978-0-387-46312-4.

[bit70033-bib-0033] Mannall, G. J. , N. J. Titchener‐Hooker , and P. A. Dalby . 2007. “Factors Affecting Protein Refolding Yields in a Fed‐Batch and Batch‐Refolding System.” Biotechnology and Bioengineering 97, no. 6: 1523–1534. 10.1002/bit.21377. 17304557

[bit70033-bib-0034] Margreiter, G. , P. Messner , K. D. Caldwell , and K. Bayer . 2008. “Size Characterization of Inclusion Bodies by Sedimentation Field‐Flow Fractionation.” Journal of Biotechnology 138, no. 3: 67–73. 10.1016/j.jbiotec.2008.07.1995. 18760314 PMC4388406

[bit70033-bib-0035] McKinney, W. 2010. Data Structures for Statistical Computing in Python.

[bit70033-bib-0036] Moran, S. D. , and M. T. Zanni . 2014. “How to Get Insight Into Amyloid Structure and Formation From Infrared Spectroscopy.” Journal of Physical Chemistry Letters 5, no. 11: 1984–1993. 10.1021/jz500794d. 24932380 PMC4051309

[bit70033-bib-0037] Ostapchenko, V. G. , M. R. Sawaya , N. Makarava , et al. 2010. “Two Amyloid States of the Prion Protein Display Significantly Different Folding Patterns.” Journal of Molecular Biology 400, no. 4: 908–921. 10.1016/j.jmb.2010.05.051. 20553730 PMC2908243

[bit70033-bib-0038] Pedregosa, F. , G. Varoquaux , A. Gramfort , et al. 2011. “Scikit‐Learn: Machine Learning In Python.” Journal of Machine Learning Research 12: 2825–2830.

[bit70033-bib-0039] Peternel, Š. , J. Grdadolnik , V. Gaberc‐Porekar , and R. Komel . 2008. “Engineering Inclusion Bodies for Non Denaturing Extraction of Functional Proteins.” Microbial Cell Factories 7, no. 1: 34. 10.1186/1475-2859-7-34. 19046444 PMC2630956

[bit70033-bib-0040] Peternel, Š. , S. Jevševar , M. Bele , V. Gaberc‐Porekar , and V. Menart . 2008. “New Properties of Inclusion Bodies With Implications for Biotechnology.” Biotechnology and Applied Biochemistry 49, no. 4: 239–246. 10.1042/BA20070140. 17708747

[bit70033-bib-0041] Reichelt, W. N. , A. Kaineder , M. Brillmann , et al. 2017. “High Throughput Inclusion Body Sizing: Nano Particle Tracking Analysis.” Biotechnology Journal 12, no. 6: 1600471. 10.1002/biot.201600471. 28301074

[bit70033-bib-0042] Rinas, U. , E. Garcia‐Fruitós , J. L. Corchero , E. Vázquez , J. Seras‐Franzoso , and A. Villaverde . 2017. “Bacterial Inclusion Bodies: Discovering Their Better Half.” Trends in Biochemical Sciences 42, no. 9: 726–737. 10.1016/j.tibs.2017.01.005. 28254353

[bit70033-bib-0043] Rolinger, L. , M. Rüdt , and J. Hubbuch . 2020. “A Critical Review of Recent Trends, and a Future Perspective of Optical Spectroscopy as Pat in Biopharmaceutical Downstream Processing.” Analytical and Bioanalytical Chemistry 412, no. 9: 2047–2064. 10.1007/s00216-020-02407-z. 32146498 PMC7072065

[bit70033-bib-0044] Rüdt, M. 2022. *Chemometrics*. Retrieved 05 August 2024. https://github.com/maruedt/chemometrics.

[bit70033-bib-0045] Rüdt, M. , T. Briskot , and J. Hubbuch . 2017. “Advances In Downstream Processing of Biologics ‐ Spectroscopy: An Emerging Process Analytical Technology.” Journal of Chromatography A 1490: 2–9. 10.1016/j.chroma.2016.11.010. 27887700

[bit70033-bib-0046] Rygula, A. , K. Majzner , K. M. Marzec , A. Kaczor , M. Pilarczyk , and M. Baranska . 2013. “Raman Spectroscopy of Proteins: A Review.” Journal of Raman Spectroscopy 44, no. 8: 1061–1076. 10.1002/jrs.4335.

[bit70033-bib-0047] Schweitzer‐Stenner, R. 2006. “Advances in Vibrational Spectroscopy as a Sensitive Probe of Peptide and Protein Structure.” Vibrational Spectroscopy 42, no. 1: 98–117. 10.1016/j.vibspec.2006.01.004.

[bit70033-bib-0048] Seabold, S. , and J. Perktold. 2010. statsmodels: Econometric and Statistical Modeling With Python. 9th Python in Science Conference (SciPy 2010), Austin.

[bit70033-bib-0049] Singh, A. , V. Upadhyay , A. Singh , and A. K. Panda . 2020. “Structure‐Function Relationship of Inclusion Bodies of a Multimeric Protein.” Frontiers in Microbiology 11: 876. 10.3389/fmicb.2020.00876. 32457730 PMC7225587

[bit70033-bib-0050] Singh, A. , V. Upadhyay , A. K. Upadhyay , S. M. Singh , and A. K. Panda . 2015. “Protein Recovery From Inclusion Bodies of *Escherichia coli* Using Mild Solubilization Process.” Microbial Cell Factories 14: 41. 10.1186/s12934-015-0222-8. 25889252 PMC4379949

[bit70033-bib-0051] Singh, S. M. , and A. K. Panda . 2005. “Solubilization and Refolding of Bacterial Inclusion Body Proteins.” Journal of Bioscience and Bioengineering 99, no. 4: 303–310. 10.1263/jbb.99.303. 16233795

[bit70033-bib-0052] Slouka, C. , J. Kopp , S. Hutwimmer , et al. 2018. “Custom Made Inclusion Bodies: Impact of Classical Process Parameters and Physiological Parameters on Inclusion Body Quality Attributes.” Microbial Cell Factories 17, no. 1: 148. 10.1186/s12934-018-0997-5. 30236107 PMC6148765

[bit70033-bib-0053] Slouka, C. , J. Kopp , O. Spadiut , and C. Herwig . 2019. “Perspectives of Inclusion Bodies for Bio‐Based Products: Curse or Blessing?” Applied Microbiology and Biotechnology 103, no. 3: 1143–1153. 10.1007/s00253-018-9569-1. 30569219 PMC6394472

[bit70033-bib-0054] Upadhyay, A. K. , A. Murmu , A. Singh , and A. K. Panda . 2012. “Kinetics of Inclusion Body Formation and Its Correlation With the Characteristics of Protein Aggregates in *Escherichia coli* .” PLoS One 7, no. 3: e33951. 10.1371/journal.pone.0033951. 22479486 PMC3315509

[bit70033-bib-0055] Valax, P. , and G. Georgiou . 1993. “Molecular Characterization of β‐lactamase Inclusion Bodies Produced In *Escherichia coli*. 1. Composition.” Biotechnology Progress 9, no. 5: 539–547. 10.1021/bp00023a014. 7764166

[bit70033-bib-0056] Vallejo, L. F. , and U. Rinas . 2004. “Strategies for the Recovery of Active Proteins Through Refolding of Bacterial Inclusion Body Proteins.” Microbial Cell Factories 3, no. 1: 11. 10.1186/1475-2859-3-11. 15345063 PMC517725

[bit70033-bib-0057] Ventura, S. , and A. Villaverde . 2006. “Protein Quality in Bacterial Inclusion Bodies.” Trends in Biotechnology 24, no. 4: 179–185. 10.1016/j.tibtech.2006.02.007. 16503059

[bit70033-bib-0058] Walsh, G. , and E. Walsh . 2022. “Biopharmaceutical Benchmarks 2022.” Nature Biotechnology 40, no. 12: 1722–1760. 10.1038/s41587-022-01582-x. PMC9735008 36471135

[bit70033-bib-0059] Walther, C. , M. C. Martinetz , A. Friedrich , et al. 2023. “Solubilization of Inclusion Bodies: Insights From Explainable Machine Learning Approaches.” Frontiers in Chemical Engineering 5: 1227620. 10.3389/fceng.2023.1227620.

[bit70033-bib-0060] Walther, C. , S. Mayer , G. Sekot , et al. 2013. “Mechanism and Model for Solubilization of Inclusion Bodies.” Chemical Engineering Science 101: 631–641. 10.1016/j.ces.2013.07.026.

[bit70033-bib-0061] Walther, C. , M. Voigtmann , E. Bruna , et al. 2022. “Smart Process Development: Application of Machine‐Learning and Integrated Process Modeling for Inclusion Body Purification Processes.” Biotechnology Progress 38, no. 3: e3249. 10.1002/btpr.3249. 35247040

[bit70033-bib-0062] Wang, L. , S. K. Maji , M. R. Sawaya , D. Eisenberg , and R. Riek . 2008. “Bacterial Inclusion Bodies Contain Amyloid‐Like Structure.” PLoS Biology 6, no. 8: e195. 10.1371/journal.pbio.0060195. 18684013 PMC2494559

[bit70033-bib-0063] Waskom, M. 2021. “Seaborn: Statistical Data Visualization.” Journal of Open Source Software 6, no. 60: 3021. 10.21105/joss.03021.

[bit70033-bib-0064] Whelan, J. , S. Craven , and B. Glennon . 2012. “In Situ Raman Spectroscopy for Simultaneous Monitoring of Multiple Process Parameters in Mammalian Cell Culture Bioreactors.” Biotechnology Progress 28, no. 5: 1355–1362. 10.1002/btpr.1590. 22740438

[bit70033-bib-0065] Wong, H. H. , B. K. O'Neill , and A. P. J. Middelberg . 1997. “Cumulative Sedimentation Analysis of *Escherichia coli* Debris Size.” Biotechnology and Bioengineering 55, no. 3: 556–564. 10.1002/(SICI)1097-0290(19970805)55:3<556::AID-BIT13>3.0.CO;2-E. 18636523

[bit70033-bib-0066] Wurm, D. J. , J. Quehenberger , J. Mildner , et al. 2018. “Teaching An Old Pet New Tricks: Tuning of Inclusion Body Formation and Properties by a Mixed Feed System In *E. coli* .” Applied Microbiology and Biotechnology 102, no. 2: 667–676. 10.1007/s00253-017-8641-6. 29159587 PMC5756567

