# Supplementary material for: The Missing Link: Connecting Cultivation Conditions and Refolding Performance via Inclusion Body Biophysical Properties
Source: Biotechnol Bioeng. 2025 Aug 5;122(11):3098–113. doi: 10.1002/bit.70033 (PMC12503015; doi:10.1002/bit.70033)
Supplement: Supplementary file 1 — Supplementary Figure 1: Schematic representation of the cultivation protocol with the different phases of the experiments and key events. Supplementary Figure 2: Workflow of IB recovery, protein solubilization, and refolding, including sampling points and analytics. Supplementary Figure 3: Pair plot of different key performance indicators of the cultivation process. Supplementary Figure 4: Scatter plot of FTIR score 1 with the two fluorescence scores. Supplementary Figure 5: Structure of anti‐desipramine antibody single chain fragment variable in‐silico predicted using AlphaFold version 2 (Jumper et al., 2021). Supplementary Table 1: Composition of buffers utilized for protL HPLC. Supplementary Table 2: Scoring of evaluated process parameters. Based on own data and literature, individual parameters were scored 1‐10 regarding “likelihood of parameter to have an influence”, “expected intensity of influence” and “controllability of the parameter”. The overall score was calculated as the product of the individual scores and the top three ranks (except for induction strength) were considered for the D‐Optimal design. Supplementary Table 3: D‐Optimal design including blocking for cultivation and refolding. Supplementary Table 4: Summary of OLS models correlating process parameters to KPIs. Empty cells correspond to a zero coefficient. Supplementary Table 5: Summary of OLS models correlating process parameters to biophysical attributes. Empty cells correspond to a zero coefficient. [file BIT-122-3098-s001.docx]

Supplementary material to

**The missing link: Connecting cultivation conditions and refolding performance via inclusion body biophysical properties**

Matthias Rüdt^1^, Aymerick Bussien^1^, Joan Cortada-Garcia^2^, Holger Morschett^2^, Hauke Holm^2^, Chiara Mazzucchelli^2^, Karlheinz Flicker^2^

**^1^ HES‑SO Valais‑Wallis, Sion, Switzerland**

**^2^ Lonza AG, Visp, Switzerland**

# Supplementary information to methods

## Strain maintenance

Strains were maintained by cryopreservation. Cultures were grown at 37 °C in 15 mL cryopreservation medium in non-baffled 100 mL shake flasks and incubated at 200 rpm orbital shaking and 25 mm shaking diameter. Exponential phase cultures were harvested upon reaching an optical density (OD_600_) of approx. 3 and glycerol was aseptically added to a final concentration of 250 g kg^‑1^. The obtained cell suspension was aliquoted. Aliquots were frozen and kept at ‑80 °C until use.

## CGE‑SDS data processing

Peaks in the 280 nm trace of CGE‑SDS between 14 min and 22 min were integrated manually with the 32 Karat software (ScieX®). Peaks before 14 min migration time were omitted as they represent ions and other small molecules. Any signal after 22 min was considered part of the baseline shift.

The product concentration $c_{P}$ in mol/L in the CGE‑SDS vial was calculated as:

|  | $\text{c}_{\text{P}}=\frac{\text{S}_{\text{P}}}{\text{S}_{\text{ref}}}\text{c}_{\text{ref}}\frac{\text{ε}_{\text{ref}}}{\text{ε}_{\text{P}}}$ | (1) |
| --- | --- | --- |

With $\text{S}_{\text{P}}$ in $s^{-1}$ being the peak area of the product, $\text{S}_{\text{ref}}$ in AU/s the peak area of the internal standard, $\text{c}_{\text{ref}} = 0.8{\mu mol L}^{-1}$ the calculated concentration of the internal standard corrected by the dilution in the CE‑SDS vial, $ɛ_{ref} = 37.47 {kL mol}^{-1}\mathrm{cm}^{-1}$ the absorption coefficient of the internal standard and $ɛ_{P} = 51.59 {kL mol}^{-1}\mathrm{cm}^{-1}$ the absorption coefficient of the product as calculated with the Expasy ProtParam tool for the reduced protein forms (Gasteiger et al., 2005).

To calculate the scFv mass fraction in the wet IB pellet $\text{χ}_{\text{3}}$ (dimensionless), $\text{c}_{\text{P}}$ was correct by the dilution steps during sample preparation, converted to a weight basis, and normalized by the mass of the solubilized pellet:

|  | $\text{χ}_{\text{3}}=\text{c}_{\text{P}}\frac{\text{V}_{\text{v}}\text{V}_{\text{s}}}{\text{V}_{\text{a}}}\frac{\text{M}_{\text{w, P}}}{\text{m}_{\text{p}}}$ | (2) |
| --- | --- | --- |

with $\text{V}_{\text{v}}$ in L being the total vial volume, $\text{V}_{\text{s}}$ in L the volume of solubilization buffer, $\text{V}_{\text{a}}$ in L the volume of solubilized IB solution added to the vial, $\text{M}_{\text{w, P}}$ in $\mathrm{kg}\mathrm{mol}^{-1}$the molar mass of the product and $\text{m}_{\text{p}}$ in kg the mass of the solubilized pellet.

The product mass $\text{m}_{\text{0,P}}$ in g in crude harvested biomass was calculated as:

|  | $\text{m}_{\text{0,P}}=\frac{\text{m}_{\text{0}}\text{+}\text{m}_{\text{1,B}}}{\text{m}_{\text{2}}}\text{m}_{\text{3}}\text{χ}_{\text{3}}$ | (3) |
| --- | --- | --- |

with $\text{m}_{\text{0}}$ in g being the mass of the wet biomass pellet, $\text{m}_{\text{1,B}}$ in g the mass of buffer added during resuspension, $\text{m}_{\text{2}}$ in g the total mass of the suspension after lysis and $\text{m}_{\text{3}}$ in g the mass of the washed wet IB pellet.

The mass fraction of product per cell dry weight pellet $\text{χ}_{\text{0}}$ (dimensionless) was calculated as:

|  | $\text{χ}_{\text{0}}=\frac{\text{m}_{\text{0,P}}}{\text{m}_{\text{X}}}$ | (4) |
| --- | --- | --- |

with $\text{m}_{\text{X}}$ in g being the cell dry mass in the suspension before cell lysis.

Finally, scFv purity in the inclusion bodies $\text{P}_{\text{P}}$ (dimensionless) was calculated as:

|  | $\text{P}_{\text{P}}=\frac{\text{S}_{\text{P}}}{\sum_{\text{i}} \text{S}_{\text{i}}}$ | (5) |
| --- | --- | --- |

with $\sum_{\text{i}} \text{S}_{\text{i}}$ in $s^{-1}$ being the sum of all relevant peak areas at 280 nm (impurities and product in the prior defined retention time range).

## IB washing and extraction in mild conditions

IB washing and extraction with mild conditions were adapted from publications and described in the following method description (Jevševar et al., 2005; Peternel et al., 2008).

The pelleted material was resuspended in 40 mL of cold deionized water and incubated for 30 min. Following the incubation period, the material was subjected to centrifugation at $1.5\cdot{10}^{4} g$ and 4 ºC for an additional 15 min. After the centrifugation, the supernatant was discarded and replenished as before. This process was repeated a second time. Two aliquots were prepared and frozen at ‑80 °C. The remaining of the washed IBs were frozen at ‑80 °C.

Pellet aliquots were thawed at room temperature and resuspended in a ratio of 1:40 (w v^‑1^) in mild detergent solution composed of 0.2 % (w v^‑1^) N‑lauroylsarcosine in 40 mM Tris/HCl at pH 8.0. This mixture was placed on a tube rotator for 24 h at room temperature to extract correctly folded scFv protein. Then, tubes were centrifuged for $4.4\cdot{10}^{3} g$ for 15 min, the supernatant was recovered for further analysis (Jevševar et al., 2005).

## Protein L HPLC

Analyses were conducted at room temperature on a 1260 Infinity II HPLC device (Agilent) equipped with a 0.1 mL CIMac™ r‑Protein L column (Sartorius) and a diode array detector (DAD) acquiring from 220 to 400 nm. Utilized buffer are described in **Supplementary Table1**. Per injection, the analytical procedure consisted of the following sequence at 1.5 mL min^‑1^ flow rate: Equilibration for 1.5 min, injection of 100 µL of 0.45 µm CA-filtered sample or standard (made by dilution of scFv standard with dialysis buffer) injection, 2.5 min of protL binding buffer, step elution of 2.5 min with protL elution buffer, re-equilibration with 2.5 min of protL binding buffer.

The scFv elution peak was integrated at 280 nm. A constant baseline was set based on the final signal intensity of the elution peak. The corresponding areas used for concentration calculations based on theoretical molar extinction coefficients of the scFv ($ɛ_{P} = 51.59{kL mol}^{-1}\mathrm{cm}^{-1}$).

# Supplementary results

## Soluble product and mild solubilization conditions

As an additional goal for this study, we also checked whether any of the designed experimental conditions led to the expression of a significant amount of soluble product. However, in no experiment, a commercially interesting amount of soluble scFv was observed. Furthermore, in the 2000s, multiple publications described processes where near native product could be extracted from IBs under mild conditions (Jevševar et al., 2005; Peternel et al., 2008). We also checked for the possibility of mild extraction in the current study. However, while we could extract DNA and components with light scattering properties from the cell pellet after cell lysis with the proposed conditions, we could not replicate the observed extraction of near-native protein (or any protein) for any of the tested fermentation conditions. This might be related to the used scFv. Since the product includes cysteines, intramolecular disulfide bridges may prevent protein extraction (see also the discussion on the Raman spectra in section 3.3 of the main manuscript). Based on our findings, we did not further investigate the mild solubilization conditions or the refolding of soluble product.

## Further analysis of spectroscopic data

While not detailed in the main text, other absorption bands seem to have a less important impact on FTIR PC 2 and 3. These impacting absorption bands are detailed below.

FTIR PC 2 is also affected by changes in the carbohydrates indicated by loadings in the murein region (Jiang et al., 2004). The loadings above 3000 cm^‑1^ could be due to variations in the OH-group concentration (e.g. varying water and carbohydrate content) as well as due to structural changes.

FTIR PC 3 potentially also collects spectral variations related to the deprotonation of carboxylic-acid-groups with two bands at 1430 cm^‑1^ and 1590 cm^‑1^ indicating symmetric and asymmetric $\mathrm{CO}O^{-}$ stretching (Jiang et al., 2004). While such a mixing is not excluded, the loadings in all amide bands support the importance of the protein backbone vibration for PC3.

# Supplementary figures


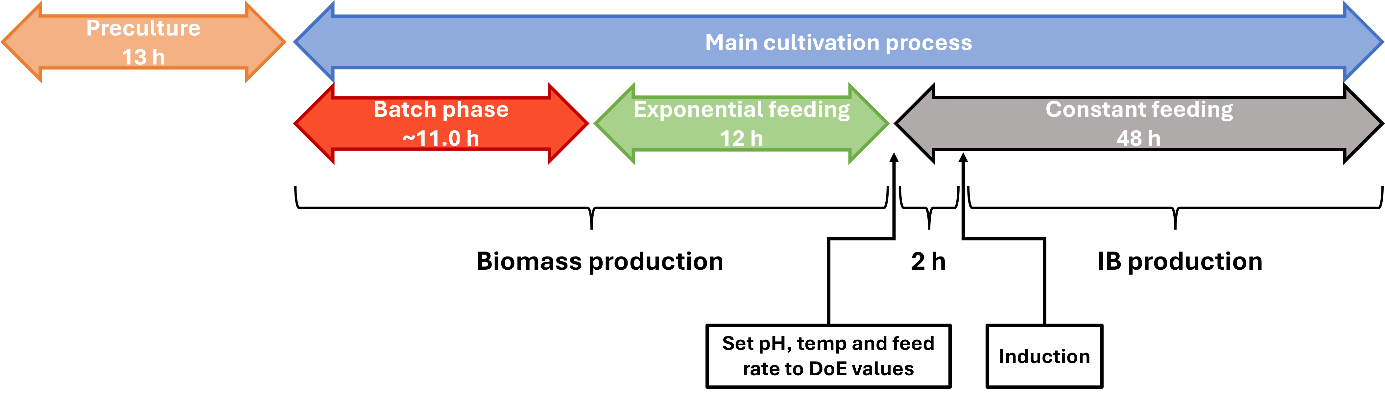


**Supplementary Figure 1:** Schematic representation of the cultivation protocol with the different phases of the experiments and key events.

**Supplementary Figure 2:** Workflow of IB recovery, protein solubilization, and refolding, including sampling points and analytics. Note: “CDW determination for DSP” refers to mass balancing only.


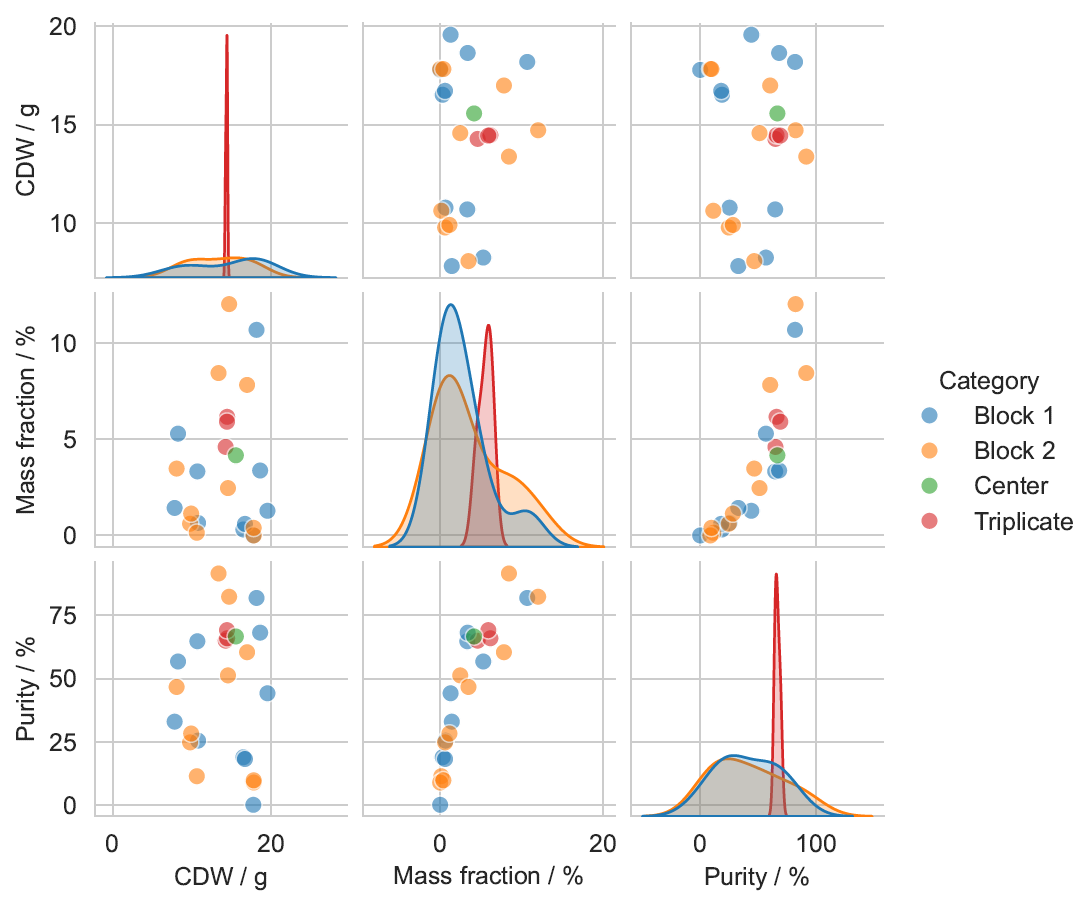


**Supplementary Figure 3:** Pair plot of different key performance indicators of the cultivation process. The data is colored by either originating from cultivation block 1 (blue) or 2 (orange). The triplicate operating conditions are marked in red and the center of the design space in green.


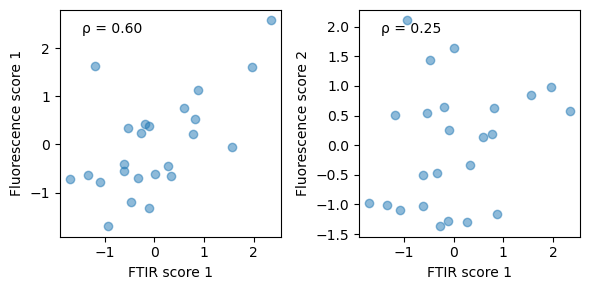


**Supplementary Figure 4:** Scatter plot of FTIR score 1 with the two fluorescence scores. The subplots also show the respective Pearson correlation coefficients. Higher fluorescence scores indicate more hydrophilic environments around tryptophan. A higher FTIR score 1 indicates less lipids and less cell debris. The positive correlation coefficients therefore support the hypothesis that the lipids are relevantly affecting the hydrophobicity around tryptophan.


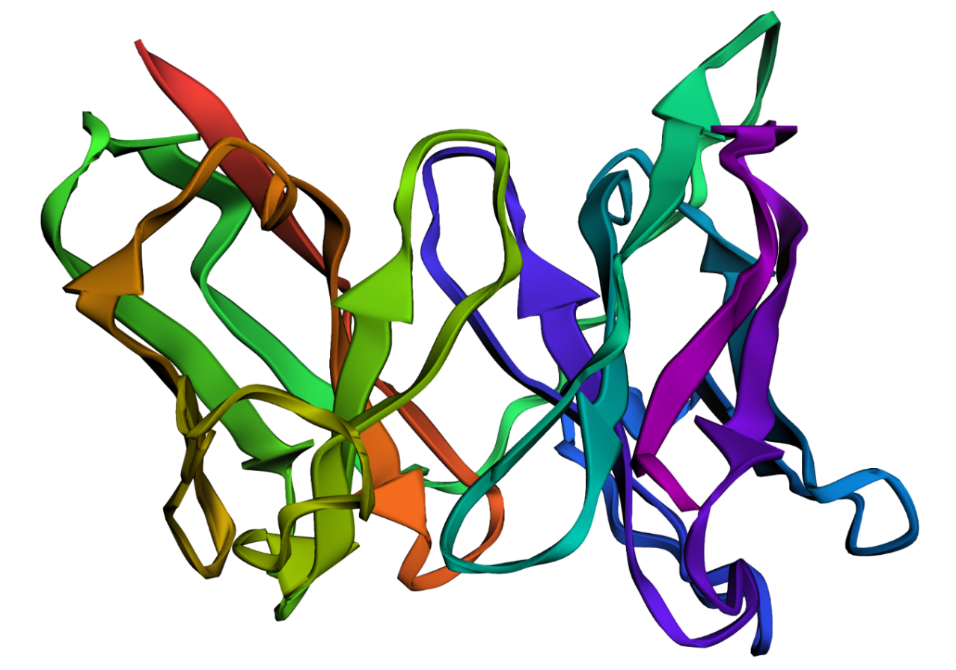


**Supplementary Figure 5:** Structure of anti-desipramine antibody single chain fragment variable *in-silico* predicted using AlphaFold version 2 (Jumper et al., 2021).

# Supplementary tables

**Supplementary Table 1:** Composition of buffers utilized for protL HPLC.

| **Buffer** | **Components** | **pH** |
| --- | --- | --- |
| ProtL elution buffer | 250 mM acetic acid | 2.5 |
| ProtL binding buffer | 50 mM Tris‑HCl, 150 mM NaCl | 7.4 |

**Supplementary Table 2:** Scoring of evaluated process parameters. Based on own data and literature, individual parameters were scored 1‑10 regarding “likelihood of parameter to have an influence”, “expected intensity of influence” and “controllability of the parameter”. The overall score was calculated as the product of the individual scores and the top three ranks (except for induction strength) were considered for the D‑Optimal design.

| **Parameter** | **Score** | | | | **References** |
| --- | --- | --- | --- | --- | --- |
|  | **Likelihood** | **Intensity** | **Controllability** | **Overall** |  |
| Temperature | 8 | 10 | 10 | 800 | Slouka et al. (2018), Slouka et al. (2019), own experiments (data not shown) |
| pH | 8 | 10 | 10 | 800 | Castellanos-Mendoza et al. (2014), Slouka et al. (2018), Slouka et al. (2019), own experiments (data not shown) |
| Induction strength | 7 | 10 | 10 | 700 | Kopp et al. (2018), Wurm et al. (2018) |
| Feeding strategy | 7 | 7 | 10 | 490 | Wurm et al. (2018) |
| DO level | 7 | 7 | 7 | 343 | Kischnick et al. (2006), Manderson et al. (2006) |
| Cell density | 3 | 5 | 5 | 75 | Slouka et al. (2019) |
| Trace elements | 1 | 5 | 3 | 15 | Dai et al. (2018) |

**Supplementary Table 3:** D‑Optimal design including blocking for cultivation and refolding.

| **ID** | **Block** | | **Coded variables** | | |
| --- | --- | --- | --- | --- | --- |
|  | **Cultivation** | **Refolding** | **pH [-]** | **T [°C]** | **Normalized feed rate [a.u.]** |
| 1 | 1 | 1 | 6.2 | 35 | 100 |
| 2 | 1 | 1 | 7.0 | 30 | 100 |
| 3 | 1 | 1 | 7.5 | 26.5 | 150 |
| 4 | 1 | 1 | 7.5 | 35 | 50 |
| 5 | 1 | 1 | 6.85 | 35 | 150 |
| 6 | 1 | 1 | 6.2 | 26.5 | 150 |
| 7 | 1 | 2 | 6.2 | 35 | 50 |
| 8 | 1 | 2 | 6.85 | 18 | 100 |
| 9 | 1 | 2 | 6.2 | 18 | 50 |
| 10 | 1 | 2 | 7.0 | 30 | 100 |
| 11 | 1 | 2 | 7.5 | 18 | 150 |
| 12 | 1 | 2 | 6.2 | 18 | 100 |
| 13 | 2 | 3 | 6.2 | 26.5 | 50 |
| 14 | 2 | 3 | 7.5 | 18 | 50 |
| 15 | 2 | 3 | 6.2 | 35 | 150 |
| 16 | 2 | 3 | 7.5 | 26.5 | 98.5 |
| 17 | 2 | 3 | 7.0 | 30 | 100 |
| 18 | 2 | 3 | 7.5 | 35 | 150 |
| 19 | 2 | 4 | 6.85 | 26.5 | 50 |
| 20 | 2 | 4 | 6.85 | 18 | 150 |
| 21 | 2 | 4 | 6.85 | 35 | 50 |
| 22 | 2 | 4 | 6.85 | 26.5 | 100 |
| 23 | 2 | 4 | 7.5 | 35 | 100 |
| 24 | 2 | 4 | 6.2 | 18 | 150 |

**Supplementary Table 4 :** Summary of OLS models correlating process parameters to KPIs. Empty cells correspond to a zero coefficient.

^‡^ normalized

| **Parameters** | **CDW in g** | **Mass fraction in %** | **Purity in %** | **Product mass in g** |
| --- | --- | --- | --- | --- |
| Intercept | 15.14 | 3.28 | 57.00 | 0.59 |
| feed_rate‡ | 4.10 | 1.33 | 6.90 | 0.30 |
| I(feed_rate‡ ** 2) | -0.76 |  | -12.88 |  |
| temperature‡ | -1.24 | 3.19 | 26.09 | 0.44 |
| I(temperature‡ ** 2) | -1.08 |  | -8.73 |  |
| pH‡ |  |  |  |  |
| I(pH‡ ** 2) |  |  |  | -0.18 |
| pH‡:feed_rate‡ |  | -1.03 | -7.07 | -0.11 |
| pH‡:temperature‡ | 0.63 |  | 4.41 |  |
| temperature‡:feed_rate‡ |  | 1.77 | 9.91 | 0.37 |
| pH‡:temperature‡:feed_rate‡ |  | -1.02 | -6.27 |  |

**Supplementary Table 5 :** Summary of OLS models correlating process parameters to biophysical attributes. Empty cells correspond to a zero coefficient.

^‡^ normalized

| **Parameter** | **Fluorescence**  **score** | | **FTIR**  **score** | | | **Raman**  **score** | | **Median**  **particle**  **diameter**  **in µm** |
| --- | --- | --- | --- | --- | --- | --- | --- | --- |
|  | **1** | **2** | **1** | **2** | **3** | **1** | **2** |  |
| R^2^ | -0.690 | -0.438 | -0.384 | -0.425 | -0.124 | -0.531 | -0.236 | -0.795 |
| Model prominence $\Delta$AICc | 1.990 | 0.230 | 0.554 | 0.029 | 0.963 | 0.374 | 0.077 | 0.068 |
| Intercept |  |  |  | -0.022 |  |  |  | -0.344 |
| pH^‡^ |  | -0.033 | -0.028 |  |  |  | -0.026 |  |
| temperature^‡^ | -0.091 | -0.034 |  | -0.028 |  | -0.041 |  | -0.058 |
| I(temperature^‡^ ** 2) | -0.156 |  | -0.058 | -0.037 |  |  |  | -0.115 |
| feed_rate^‡^ |  | -0.022 |  |  |  | -0.044 |  |  |
| I(feed_rate^‡^ ** 2) | -0.174 |  | -0.070 |  |  |  |  |  |
| pH^‡^:feed_rate^‡^ |  |  |  | -0.020 |  |  |  | -0.054 |
| pH^‡^:temperature^‡^ | -0.101 |  |  |  | -0.015 |  |  | -0.038 |
| temperature^‡^:feed_rate^‡^ | -0.120 |  |  |  |  | -0.042 |  | -0.052 |
| pH^‡^:temperature^‡^:feed_rate^‡^ |  |  |  |  |  |  | -0.027 | -0.057 |

# References

Castellanos-Mendoza, A., Castro-Acosta, R. M., Olvera, A., Zavala, G., Mendoza-Vera, M., García-Hernández, E.,…Valdez-Cruz, N. A. (2014). Influence of pH control in the formation of inclusion bodies during production of recombinant sphingomyelinase-D in *Escherichia coli*. *Microbial Cell Factories*, *13*(1), 137. <https://doi.org/10.1186/s12934-014-0137-9>

Dai, X., Zhu, M., Warren, M., Balakrishnan, R., Okano, H., Williamson, J. R.,…Hwa, T. (2018). Slowdown of translational elongation in *Escherichia coli* under hyperosmotic stress. *mBio*, *9*(1), 10.1128/mbio.02375-02317. <https://doi.org/10.1128/mbio.02375-17>

Gasteiger, E., Hoogland, C., Gattiker, A., Duvaud, S. e., Wilkins, M. R., Appel, R. D., & Bairoch, A. (2005). Protein identification and analysis tools on the ExPASy server. In J. M. Walker (Ed.), *The Proteomics Protocols Handbook* (pp. 571-607). Humana Press. <https://doi.org/10.1385/1-59259-890-0:571>

Jevševar, S., Gaberc-Porekar, V., Fonda, I., Podobnik, B., Grdadolnik, J., & Menart, V. (2005). Production of nonclassical inclusion bodies from which correctly folded protein can be extracted. *Biotechnology Progress*, *21*(2), 632-639. <https://doi.org/10.1021/bp0497839>

Jiang, W., Saxena, A., Song, B., Ward, B. B., Beveridge, T. J., & Myneni, S. C. B. (2004). Elucidation of functional groups on gram-positive and gram-negative bacterial surfaces using infrared spectroscopy. *Langmuir*, *20*(26), 11433-11442. <https://doi.org/10.1021/la049043>+

Jumper, J., Evans, R., Pritzel, A., Green, T., Figurnov, M., Ronneberger, O.,…Hassabis, D. (2021). Highly accurate protein structure prediction with AlphaFold. *Nature*, *596*(7873), 583-589. <https://doi.org/10.1038/s41586-021-03819-2>

Kischnick, S., Weber, B., Verdino, P., Keller, W., Sanders, E. A., Anspach, F. B.,…Suck, R. (2006). Bacterial fermentation of recombinant major wasp allergen antigen 5 using oxygen limiting growth conditions improves yield and quality of inclusion bodies. *Protein Expression and Purification*, *47*(2), 621-628. <https://doi.org/10.1016/j.pep.2006.01.009>

Kopp, J., Slouka, C., Strohmer, D., Kager, J., Spadiut, O., & Herwig, C. (2018). Inclusion body bead size in *E. coli* controlled by physiological feeding. *Microorganisms*, *6*(4). <https://doi.org/10.3390/microorganisms6040116>

Manderson, D., Dempster, R., & Chisti, Y. (2006). A recombinant vaccine against hydatidosis: production of the antigen in *Escherichia coli*. *Journal of Industrial Microbiology and Biotechnology*, *33*(3), 173-182. <https://doi.org/10.1007/s10295-005-0046-3>

Peternel, Š., Grdadolnik, J., Gaberc-Porekar, V., & Komel, R. (2008). Engineering inclusion bodies for non denaturing extraction of functional proteins. *Microbial Cell Factories*, *7*(1), 34. <https://doi.org/10.1186/1475-2859-7-34>

Slouka, C., Kopp, J., Hutwimmer, S., Strahammer, M., Strohmer, D., Eitenberger, E.,…Herwig, C. (2018). Custom made inclusion bodies: impact of classical process parameters and physiological parameters on inclusion body quality attributes. *Microbial Cell Factories*, *17*(1), 148. <https://doi.org/10.1186/s12934-018-0997-5>

Slouka, C., Kopp, J., Spadiut, O., & Herwig, C. (2019). Perspectives of inclusion bodies for bio-based products: curse or blessing? *Applied Microbiology and Biotechnology*, *103*(3), 1143-1153. <https://doi.org/10.1007/s00253-018-9569-1>

Wurm, D. J., Quehenberger, J., Mildner, J., Eggenreich, B., Slouka, C., Schwaighofer, A.,…Spadiut, O. (2018). Teaching an old pET new tricks: tuning of inclusion body formation and properties by a mixed feed system in *E. coli*. *Applied Microbiology and Biotechnology*, *102*(2), 667-676. <https://doi.org/10.1007/s00253-017-8641-6>
